# Computational identification of new potential transcriptional partners of ERRα in breast cancer cells: specific partners for specific targets

**DOI:** 10.1038/s41598-022-07744-w

**Published:** 2022-03-09

**Authors:** Catherine Cerutti, Ling Zhang, Violaine Tribollet, Jing-Ru Shi, Riwan Brillet, Benjamin Gillet, Sandrine Hughes, Christelle Forcet, Tie-Liu Shi, Jean-Marc Vanacker

**Affiliations:** 1 Institut de Génomique Fonctionnelle de Lyon, Université de Lyon, Université Lyon 1, CNRS UMR5242, Ecole Normale Supérieure de Lyon, 32-34 Avenue Tony Garnier, 69007 Lyon, France; 2 The Center for Bioinformatics and Computational Biology, Shanghai Key Laboratory of Regulatory Biology, Institute of Biomedical Sciences and School of Life Sciences, East China Normal University, Shanghai, China

**Keywords:** Breast cancer, Cancer, Computational biology and bioinformatics, Computational models

## Abstract

Estrogen related receptors are orphan members of the nuclear receptor superfamily acting as transcription factors (TFs). In contrast to classical nuclear receptors, the activities of the ERRs are not controlled by a natural ligand. Regulation of their activities thus relies on availability of transcriptional co-regulators. In this paper, we focus on ERRα, whose involvement in cancer progression has been broadly demonstrated. We propose a new approach to identify potential co-activators, starting from previously identified ERRα-activated genes in a breast cancer (BC) cell line. Considering mRNA gene expression from two sets of human BC cells as major endpoint, we used sparse partial least squares modeling to uncover new transcriptional regulators associated with ERRα. Among them, *DDX21*, *MYBBP1A*, *NFKB1*, and *SETD7* are functionally relevant in MDA-MB-231 cells, specifically activating the expression of subsets of ERRα-activated genes. We studied SET7 in more details and showed its co-localization with ERRα and its ERRα-dependent transcriptional and phenotypic effects. Our results thus demonstrate the ability of a modeling approach to identify new transcriptional partners from gene expression. Finally, experimental results show that ERRα cooperates with distinct co-regulators to control the expression of distinct sets of target genes, thus reinforcing the combinatorial specificity of transcription.

## Introduction

In eukaryotes, regulation of gene expression relies on a combinatorial interplay between transcriptional regulators (TRs) including DNA-binding transcription factors (TFs) and non-DNA binding co-activators or co-repressors. Among non-DNA-binding co-regulators, those involved in histone modifications are of importance to control chromatin accessibility and the dynamics of the transcriptional process^1^. The coordinated activity of all these cooperating factors results in specific spatio-temporal effects on target gene expression^2^.

Searching potential TFs by identifying TF-binding sites in pre-defined regions from the transcription start site (TSS) has often been used to unveil cooperative binding of TFs^3–5^. However, presence of binding sites is not sufficient to predict actual binding of TFs necessary for cooperation. Chromatin immunoprecipitation sequencing (ChIP-seq) studies provide actual genomic locations of TF DNA-binding. It however still remains challenging to determine whether these binding events are functional or incidental and whether they function in conjunction with other TFs nearby or at a distance. Several experimental approaches can demonstrate pairwise interactions at the protein level between TFs or between TF and non-DNA binding co-activator^6–8^. However simultaneous cooperative recruitment of more than two transcriptional partners may occur and is difficult to demonstrate experimentally. Various in silico methods have been proposed to infer either gene regulatory networks using dynamic or static mRNA gene expression^9–12^ or transcriptional regulatory networks using mRNA and protein data to suggest direct relationships between regulators and target genes^13–15^. Such methods were developed in various biological contexts including breast cancer (BC) for subtypes identification^16,17^. Moreover, uncovering combinations of regulators that could be simultaneously or sequentially recruited remains to be achieved.

The estrogen-related receptors (ERRα, β and γ in mammals) are a family of orphan nuclear receptors acting as TFs. They are expressed in several tissues and display various physiological and pathological functions^18–21^. In particular, ERRα is involved in energy metabolism, osteogenesis and tumorigenesis^22–24^. In cancer, ERRα has been shown to control several parameters, including proliferation and cell migration^20,25,26^. All ERRs include a DNA-binding domain responsible for the specific binding to TCAAGGTCA sequence (ERR response element, ERRE). They activate target genes in a ligand-independent manner in the presence of co-activators. Several co-regulators of ERRα have been identified in the frame of its involvement in energy metabolism, such as PGC-1α^27^. In contrast, RIP140 can act as an ERRα corepressor, depending on the regulatory elements in target promoters^28^. ERRα regulation of oxidative metabolism is also repressed by NCoR1 in skeletal muscle therefore competing with PGC-1α^29^. The regulation of cell migration by ERRα does not depend on PGC-1α^26^ indicating that ERRα modulates different gene repertoires, depending on the co-regulator with which it interacts. In this line, our team previously identified the histone lysine specific demethylase 1 (LSD1) as an important co-regulator of ERRα in BC cell migration^30^. In addition, NRF1 was found associated with the ERRα-LSD1 complex and recruited at the TSS of positive ERRα-LSD1 targets related to cell invasion^31^. However, all these factors were identified one at a time in various cell or animal models either in a molecular or a gene approach, and we are still lacking a more global view.

In this work, we focused on the functional cooperation of ERRα with both TFs and non-DNA binding co-activators in BC cells. Taking mRNA gene expression as major endpoint, quantitative statistical modeling of ERRα target gene expression from TR expression was performed to uncover new transcriptional co-activators of ERRα. Our results highlighted specific TRs associated with the ERRα-encoding gene *ESRRA* in the expression models of ERRα-activated genes across various BC cells. Among them, *DDX21*, *MYBBP1A*, *NFKB1*, and *SETD7* were validated in MDA-MB-231 cells as modulators of distinct sets of ERRα-activated genes. These results demonstrate the ability of the modeling approach to identify new transcriptional partners. Each combination of ERRα and co-activators regulates specific sets of ERRα target genes, thus reinforcing the combinatorial specificity of transcription.

## Results

### Genes submitted to the modeling process

**Figure 1:**
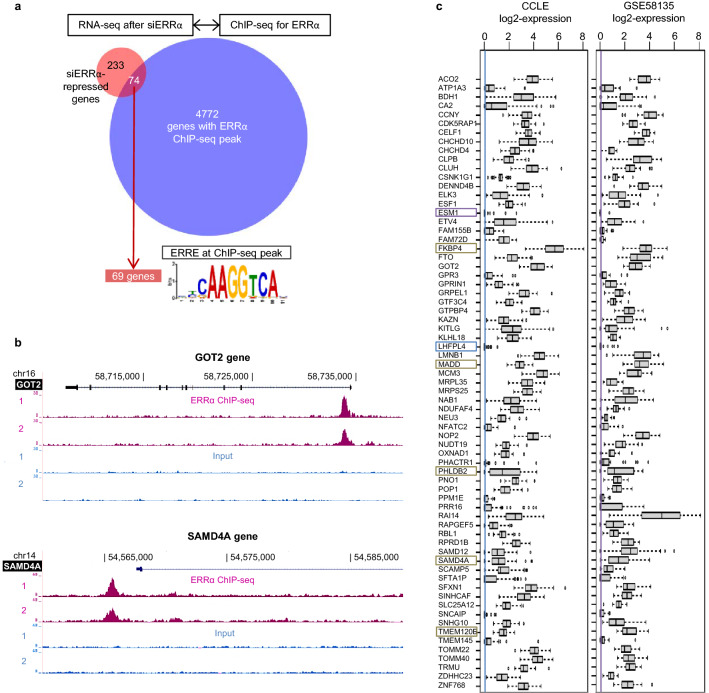
Direct ERRα-activated genes in MDA-MB-231 cells. (**a**) Venn diagram showing the amount of genes displaying reduced expression upon siERRα treatment and associated with a ChIP-seq peak for ERRα. The ERR response element (ERRE, JASPAR motif MA0592.3) was detected at ChIP-seq peak (± 250 bp around peak) by the FIMO tool of the MEME-Suite for 93% of the identified ERRα-activated genes, which were taken as direct ERRα-activated targets. (**b)** Graphs obtained from the UCSC browser showing the two replicates of ERRα ChIP-seq and input signals for two genes displayed as examples. (**c)** Expression of the 69 identified ERRα-activated genes in the BC cells of CCLE and GSE58135 datasets. Expression is log2 of upper-quartile normalized TPM (for CCLE) or FPKM (for GSE58135) values. In each dataset, genes are ordered according to decreasing mean expression value. Color rectangles indicate removed genes due to low expression (colored line) or not expressed in at least one cell line of the dataset.

To determine TRs that would be associated to ERRα activity, we focused on ERRα direct positive target genes. To establish this gene list, we first performed a ChIP-seq analysis in MDA-MB-231 cells (Figure S1). This revealed 5205 significant reproducible peaks associated to 4846 distinct genes identified by nearest TSS (data available in Table S1). We next compared this list to the 307 genes previously identified by RNA-seq in MDA-MB-231 cells as positively modulated by ERRα^30^. 74 genes (24.1%) were found associated to a ChIP-seq peak for ERRα among which 69 altogether displayed a consensus ERRE motif at the peak summit (Fig. 1a, b). Using two public expression datasets obtained in BC cells including MDA-MB-231 cells for both, we found that these genes showed variable expression levels and variable dispersion of expression across cell lines (Fig. 1c). These 69 genes, hereafter referred to as ERRα-activated genes, were used for further modeling of their expression.

**Figure 2:**
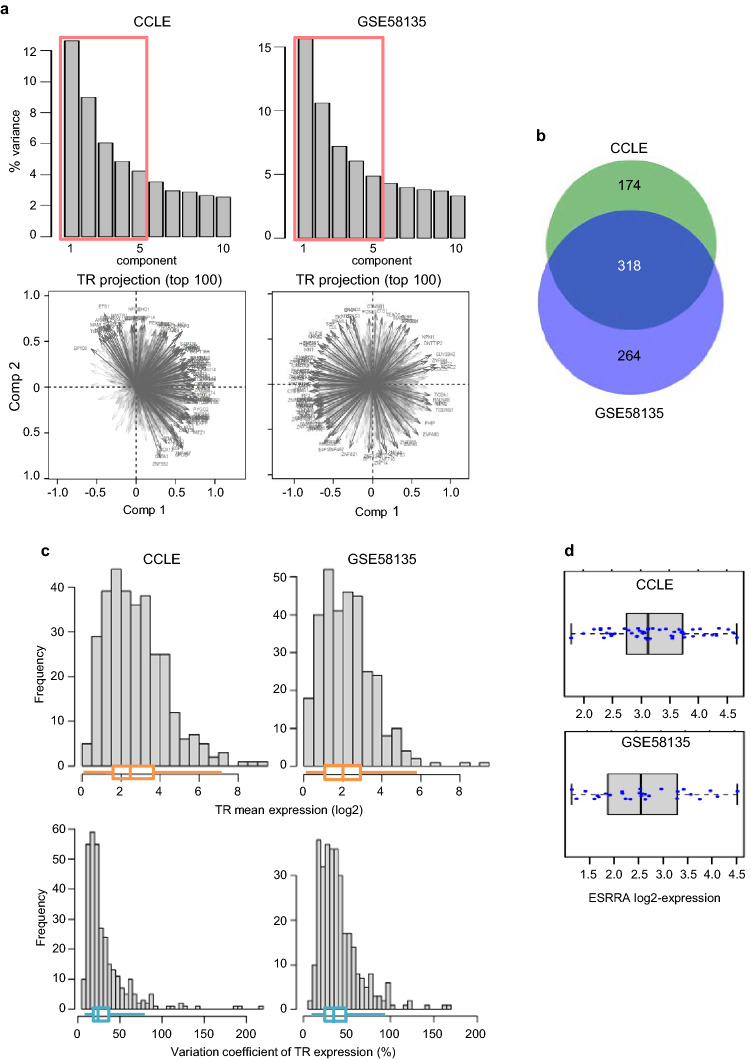
Identification of a reduced set of TRs. (**a)** PCA (FactoMineR R package) on TR log2-expression across cell lines in each dataset. In the upper panels the scree plots show the selection of the 5 first principal components (37 to 44% of the total variance). The lower panels show the projection of the 50 best TRs taken as variables on the 2 first principal axes in each dataset. For each dataset, absolute value of correlation coefficient > 0.5 with each of the axes was taken as meaningful. (**b)** Venn diagram showing the selected TRs common to the 2 datasets. **(c)** Distributions of mean expression and of expression variation coefficient of the 318 selected TRs across cells in each dataset associated with boxplots. They show moderate expression of the majority of TRs with variation coefficient across cells mostly between 20 and 50% (inter-quartile range). (**d)** Boxplots showing ESRRA expression and variability in the BC cells of CCLE and GSE58135 datasets. (**c, d)** Expression is log2 of upper-quartile normalized TPM (for CCLE) or FPKM (for GSE58135) values.

We next establish a list of TRs expressed in BC cells taken as explanatory variables in the modeling approach. To this end, using expression-based criteria and principal component analysis, we identified relevant TRs from a comprehensive set of 2175 TRs collected from several public databases. Among the 1308 TRs that passed expression-based criteria in the two studied datasets, those best correlated with each of the first five principal components, *i.e.* the most expression-varying TRs across cells, were identified for each dataset. As a result, 318 TRs common to the two datasets were pre-selected for model computation (Fig. 2a, b and Table S2). Most of these TRs exhibited moderate expression but sufficient expression variability across BC cells as shown by variation coefficient higher than 23% and 35% for more than half of them in CCLE and GEO-GSE58135 data respectively (Fig. 2c). Among them, *ESRRA* showed quite suitable expression and expression variability across cells in both datasets for use in the modeling approach (Fig. 2d).

### Identification of TRs associated to *ESRRA* by statistical modeling of gene expression

#### Characteristics of computed models

**Figure 3:**
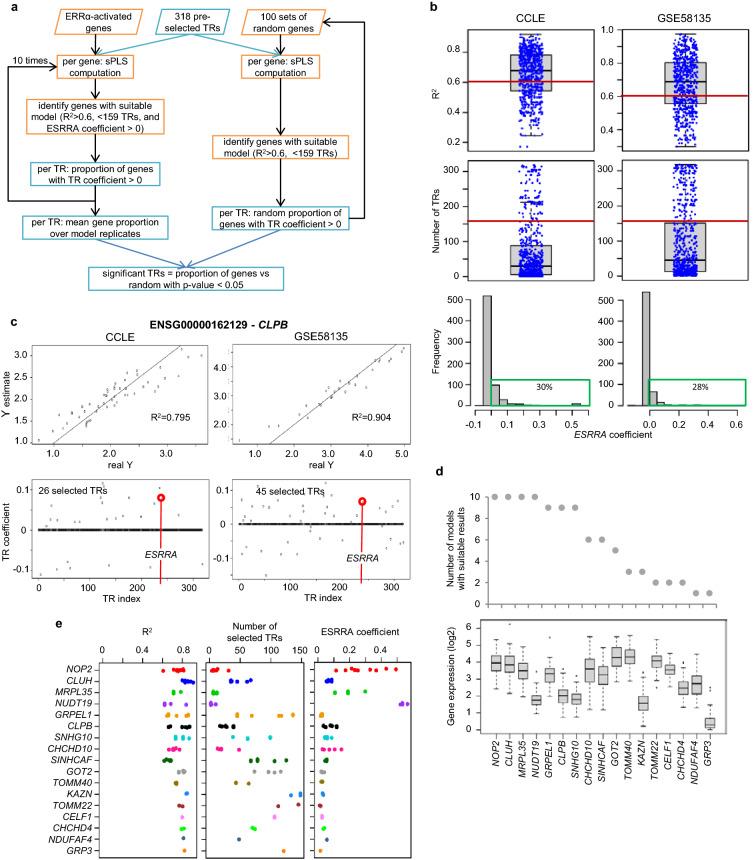
Model features across all ERRα-activated genes and all replicated analyses using two expression datasets. (**a)** Flowchart of the modeling procedure used to select TRs. (**b)** Boxplots showing individual model R-squared values and the number of TRs selected by the model, i.e. with non-0 coefficient, among 318 ones in each model computed 10 times for 68 and 63 genes with the CCLE and GSE58135 dataset respectively. Red lines indicate the thresholds used for further selection (R2 > 0.6 and number of selected TRs > 159). The lower panel shows histograms of ESRRA coefficient values in models. Most of the values are in the interval ]−0.05, 0] leading to 206/680 (30%) and 177/630 (28%) models including ESRRA with a positive coefficient for CCLE and GSE58135 data respectively. The green rectangles indicate positive ESRRA coefficients. **(c)** Example of model results for one gene with the two datasets: scatterplots of predicted vs true gene expression and values of TR coefficients given by the model. Data are log2-transformed and the identity line is shown. The TRs selected in one model have non-0 coefficient values and include ESRRA with a positive coefficient. (**d)** ERRα-activated genes with suitable expression model in CCLE dataset: number of model replicates giving suitable results and expression of these 17 genes given as boxplot across the 51 breast cancer cells. (**e)** Model characteristics across the 10 replicates for the 17 ERRα-activated genes with at least one suitable expression model in CCLE dataset.

Sparse PLS (sPLS) models were computed for individual ERRα-activated genes that passed the expression criteria in the dataset: 68/69 genes for the CCLE data in 51 BC cells and 63/69 genes for the GEO-GSE58135 data in 28 BC cells (Fig. 1c, Table S3). The removed genes displayed either median expression value at 0 or some missing values. Models used the 318 short-listed TRs that all passed the expression criteria. Model computation was replicated 10 times for each gene according to the flowchart shown in Fig. 3a. Half of the sPLS models gave R-squared values > 0.68 in both datasets and included a reduced number of TRs (median at 30 for CCLE and 45 for GSE58135) (Fig. 3b). Only about 30% of the models included *ESRRA* as significant TR (non-0 coefficient) (Fig. 3b). The latter result may be a simple consequence of some redundancy between TRs that regulate each other’s expression. Other characteristics of the computed models (lambda value, number of latent components, and residual variance) are given in Figure S2-a.

Computed models fulfilling quality criteria (R^2^ > 0.6, number of TRs < 159, and ESRRA coefficient > 0) were further examined. Figure 3c gives an example of such models obtained for one ERRα-activated gene using the two datasets. It shows the quality of the prediction of gene expression and it can be observed that the *ESRRA* coefficient is one the highest ones among the 318 TRs.

The ERRα-activated genes that gave suitable models including *ESRRA* as a positive variable in at least one replicate were identified. For the GSE58135 dataset, only 10 ERRα-activated genes fulfilled this criterion. For the CCLE dataset, 17 ERRα-activated genes were uncovered including 9 of the 10 ones stated above (Fig. 3d). For these 17 genes, the computed models exhibited some variation across replicates (Fig. 3e). They included mostly less than 50 selected TRs, and R^2^ ranged between 0.6 and 0.85. The ESRRA coefficient was clearly different between genes but quite stable across replicates. In addition, it consistently varied inversely to the number of selected TRs but independently of R^2^, and R^2^ was not related to the number of selected TRs (Figure S2-b).

#### Best TRs associated to *ESRRA*

**Figure 4:**
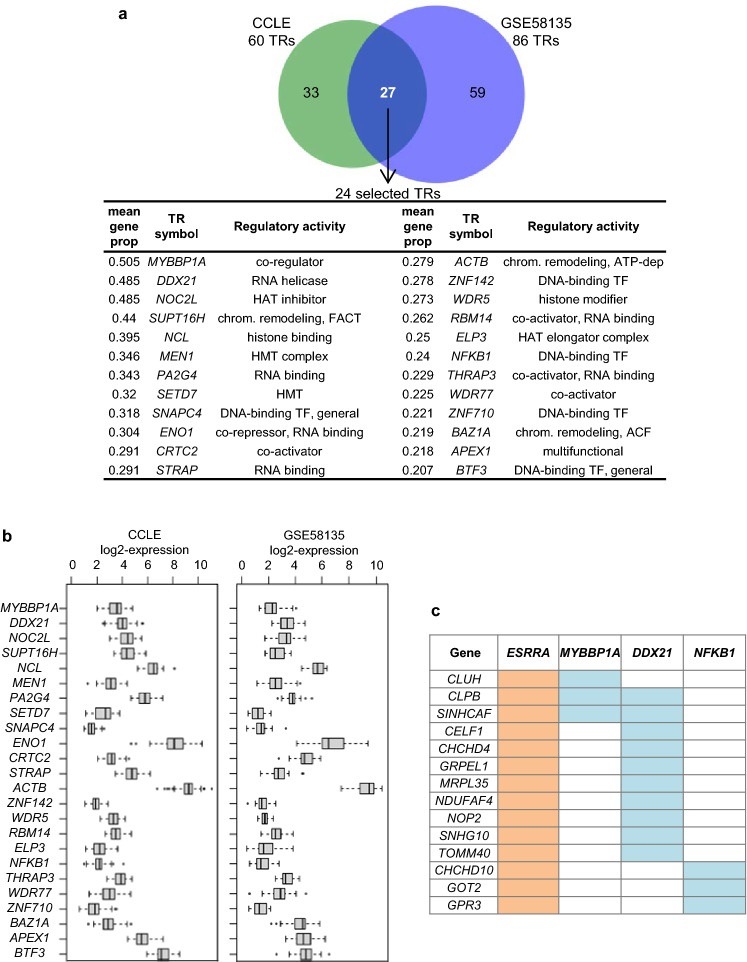
Transcriptional regulators associated with ESRRA in sPLS expression models of ERRα-activated genes. (**a)** Venn diagram of TRs with model coefficient > 0 and *p*-value < 0.05 identified from the ERRα-activated genes with reliable sPLS models in CCLE and GSE58135 datasets. The 24 selected TRs commonly identified in the two datasets are ordered according to the mean proportion of genes (mean gene prop, > 0.2) over the CCLE and the GSE58135 datasets for which the TR has positive coefficient in the 10 model replicates computed with one dataset. HAT: histone acetyltransferase, FACT: facilitates chromatin transcription, HMT: histone methyltransferase, chrom: chromatin, ACF: ATP-dependent chromatin assembly factor, TF: transcription factor. (**b)** Expression boxplots for the 24 selected TRs (same order as table above). Expression is upper-quartile normalized by sample and log2-transformed. (**c)** Genes including at least one of *MYBBP1A, DDX21* or *NFKB1* as selected TR in addition to *ESRRA* in their sPLS models.

Significant TRs were identified in each dataset from suitable expression models. They were sorted according to the proportion of ERRα-activated genes including them in their models. As shown in Fig. 4a, among the TRs identified in both datasets, 24 ones detected for > 20% of the genes as a mean were selected. All of these 24 selected TRs had high expression levels in both datasets (mean expression over BC cells > 75th genome percentile) (Fig. 4b).

Interestingly, these TRs included a majority of non-DNA-binding factors and only three DNA-binding TFs, notably *NFKB1* encoding a subunit of the NF-κB protein complex. *MYBBP1A* and *DDX21* as well as *NFKB1* were selected for further investigation in MDA-MB-231 cells. The 17 ERRα-activated genes with suitable models were then filtered according to the presence of these TRs in their models leading to 14 genes (Fig. 4c). Detailed features of suitable computed models for the 17 ERRα-activated genes are in Table S4.

### Candidate TRs modulate expression of ERRα-activated genes

Among the 14 ERRα-activated genes, we picked two genes associated to one of the three candidate TRs or to the only TR pair, resulting in seven genes that were submitted to experimental validation in MDA-MB-231 cells. As negative controls, we used four genes regulated by ERR (as evidenced by our previous RNA-seq analysis) but not associated to any of the selected TRs. All of these 11 genes had RPKM expression > 5 in MDA-MB-231 cells (Figure S3).

**Figure 5:**
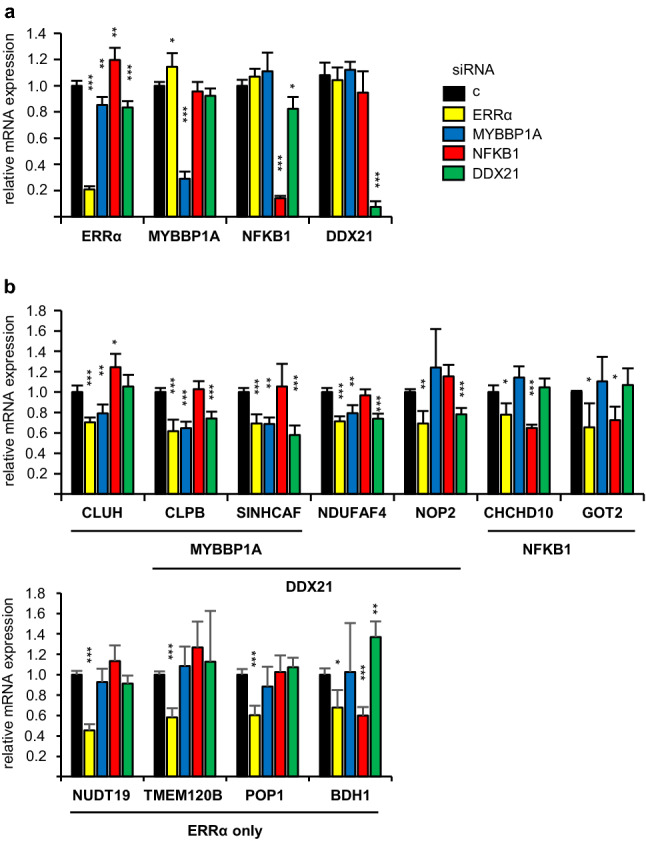
Regulation of gene expression by ERRα and suspected co-regulators. (**a, b)** MDA-MB-231 cells transfected with siRNAs directed against the indicated transcription factors were analyzed for expression of the indicated genes by RT-qPCR. Results are presented relative to control conditions with bars representing mean + /- sem of three independent experiments performed in triplicate. (**a)** Validation of siRNA effect on their corresponding direct targets. (**b)** Analysis of ERRα target gene. As evaluated by t-test, variations are not significant unless indicated by ***p < 0.001, **p < 0.01, *p < 0.05.

ERRα binding to the genomic sequences at ChIP-seq peak was first confirmed in MDA-MB-231 cells by independent ChIP-qPCR experiments (Figure S4-a). We next analyzed by RT-qPCR the effect of efficient siRNA-mediated inactivation of ERRα or of each of the three TRs (Fig. 5a, b). As expected, depletion of ERRα reduced the expression of all 7 ERRα-TR associated genes. In agreement with our approach, inactivation of NFKB1, MYBBP1A or DDX21 also decreased the expression of their corresponding associated genes, suggesting that our approach did not produce any false positive association. The expression of all four negative controls was also decreased by ERRα depletion but not by those of the candidate TRs with the exception of BDH1, reduced upon NFKB1 inactivation. Together with the unpredicted effect of MYBBP1A on *NDUFAF4*, this suggests the existence of false negative target genes within a given ERRα-TR association. The stringent criteria we used in our approach may account for the fact that false negative genes escaped our model TR selection. Similar results were observed in SKBr3 and MCF7 BC cells contrary to HeLa cells, a cervical cancer cell line (Figure S4-b).

### SET7 as an ERRα-dependent transcriptional co-activator

**Figure 6:**
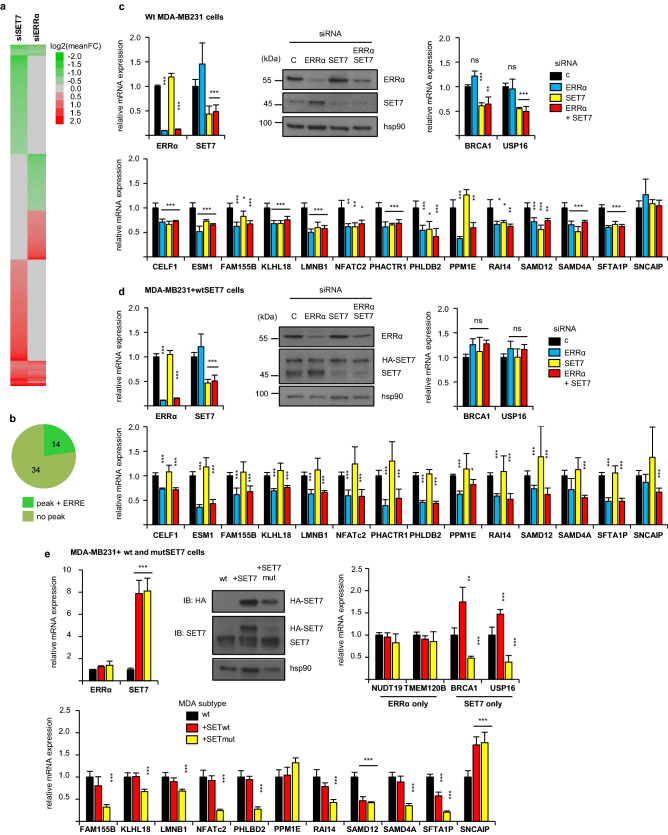
Common transcriptional targets of SET7 and ERRα. (**a)** Heatmap of the 1407 genes over- or under-expressed upon siSET7 or siERRα using mean log2 fold change (FC) over 2 distinct siRNAs (scale indicated). Mean FC is set to 1 for genes with no significant expression change. (**b)** Pie chart summarizing the number of genes with reduced expression upon siSET7 and siERRα that were associated with a ChIP-seq peak for ERRα. The 14 ERRα-SET7 activated genes present an ERRE motif at ChIP-seq peak summit. (**c)** Wild-type MDA-MB-231 cells transfected with siRNAs directed against SET7 and/or ERRα were analyzed for expression of the indicated genes by RT-qPCR. Expression of SET7 and ERRα proteins was also analyzed by Western blot (marker size is indicated) using hsp90 as a loading control. RT-qPCR results are presented relative to control conditions with bars representing mean + /- sem of three independent experiments performed in triplicate. (**d)** Same experiments performed in HA-SET7-overexpressing MDA-MB-231 cells (MDA-MB231 + SET7). (**e)** Same experiment performed in HA-SET7 (mutant or wild type) overexpressing SET7. As evaluated by t-test, variations are not significant unless indicated by ***p < 0.001, **p < 0.01, *p < 0.05. Uncropped Western blot images are presented on Figure S9.

To further validate our approach, we chose to study more thoroughly the association of ERRα with SET7, which was selected in our sPLS modeling and has been shown to be associated with cancer progression^32–36^. The effect of ERRα in BC progression might thus be partly explained by its combined transcriptional activity with SET7. To this end, we first performed an RNA-seq analysis after siRNA-mediated SET7 inactivation in MDA-MB-231 cells. This identified 503 genes whose expression was decreased upon by SET7 depletion. Comparison with our previous ERRα RNA-seq data revealed a reduced expression after ERRα inactivation for 48 of them (Fig. 6a), out of which 14 were associated with one or more ERRα ChIP-seq peak(s) (Fig. 6b). Only one of these genes (*CELF1*) belonged to the above-mentioned 17 ERRα-activated genes with suitable models (Fig. 3d, e). This low number may be due to the presence of false negatives in sPLS models. In order to overcome this limitation, we analyzed all of the 14 ERRα-activated genes activated by SET7.

ERRα binding at ChIP-seq peaks was first confirmed in MDA-MB-231 cells by independent ChIP-qPCR experiments (Figure S5-a). The effect of ERRα and SET7 on target genes expression was next analyzed by RT-qPCR. With the exception of *PPM1E* and *SNCAIP*, the expression of all these genes was decreased upon siRNA-mediated inactivation of ERRα or SET7 (Fig. 6c), confirming our RNA-seq analyses. Interestingly, simultaneous ERRα and SET7 silencing did not further decrease target gene expression relative to single factor inactivation, suggesting that both factors act in the same pathway. Similar results were observed for siRNA experiments in SKBr3 and HeLa cells, but surprisingly not in MCF7 cells (Figure S5-b).

We further tackled the question of ERRα and SET7 functional interactions by performing rescue experiments. To this end, we generated MDA-MB-231 cell populations overexpressing HA-SET7 in a mutant version that escapes our siRNA (Fig. 6d). In these cells, the expression of the 14 ERRα-SET7 targets is still reduced upon ERRα depletion. As expected, siRNA-mediated depletion of the endogenous SET7 moiety did not result in any reduction of target gene expression, in contrast to the situation in wild-type cells. Strikingly, the HA-SET7 transgene is completely unable to rescue target gene expression when ERRα is depleted together with endogenous SET7 (Fig. 6d). As controls, we used *BRCA1* and *USP16*, identified by RNA-seq as SET7-, but not ERRα-, targets. Expression of these two genes was sensitive to SET7-depletion in wild type-, but not in HA-SET7 overexpressing cells, whatever the ERRα status (Fig. 6c, d). In addition, we generated MDA-MB-231 cells that overexpress wild type or methyltransferase-dead SET7 mutant (Fig. 6e). Nearly all ERRα-SET7 targets were down-regulated in the presence of mutated SET7, but not in the presence of wild type SET7.

**Figure 7:**
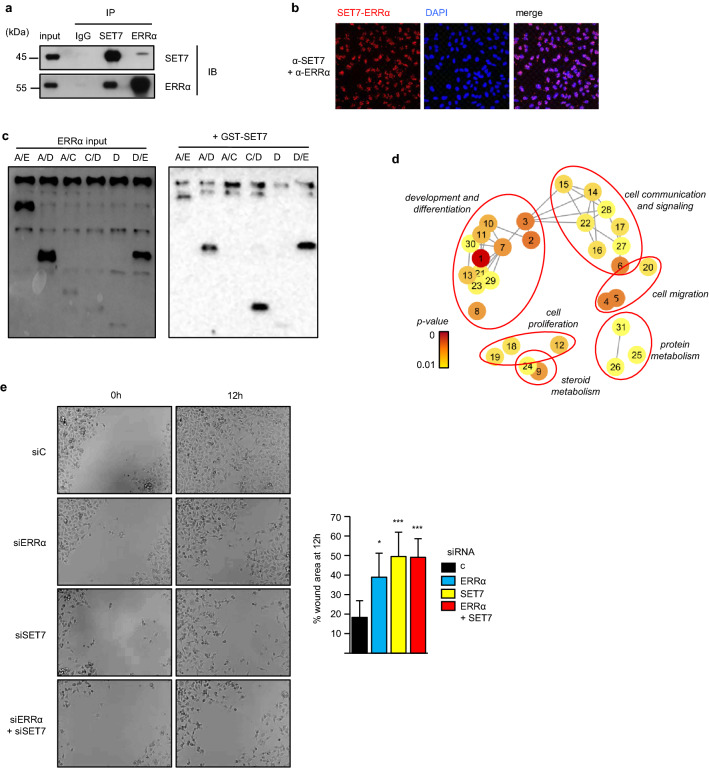
SET7 and ERRα are involved in cell invasion. (**a)** Co-immunoprecipitation of endogenous proteins in MDA-MB-231 cells with anti-SET7 or anti-ERRα antibodies and rabbit IgG used as a control. IP: immunoprecipitation, IB: immunoblotting. Proximity ligation assay (PLA) used to detect interaction of endogenous SET7 and ERRα in MDA-MB231 cells. Cells were counterstained with DAPI. See also Figure S5-c for PLA controls. (**c)** Indicated ERRα moiety were produced in vitro and identified on the left panel. Same ERRα moiety were hybridized on GST-SET7. (**d)** ERRα/SET7 regulated genes were analyzed by Gene Ontology (GO). After elimination of redundant terms, network of enriched GO terms obtained by REVIGO software (grouped according to semantic similarity) is shown. Colors indicate *p*-value. GO terms are coded by number (see Figure S6-a for correspondence with GO terms). **(e)** Confluent layers of MDA-MB-231 cells transfected with the indicated siRNA were scratch-wounded, phase contrast microphotographs were taken at the indicated times after wounding (left panels). Quantification (right panel) is displayed as percentage of remaining cell-free space at 12 h. Data are means of three independent experiments (3 fields per experiment) + /- sem. Significance was evaluated by t-test, with ***p < 0.005, *p < 0.05. Uncropped Western blot images are presented on Figure S9.

Altogether, this shows that SET7 requires ERRα to transactivate ERRα-SET7 common target genes. Consistently, a physical interaction between the two factors was identified by co-immunoprecipitation, which occurs in the nucleus as indicated by proximity ligation assays (Fig. 7a, b). In addition, in vitro immunoprecipitation identified the D domain in ERRαas responsible for the SET7 interaction (Fig. 7c). The genes commonly regulated by ERRα and SET7 were submitted to Gene Ontology (GO) analysis, which showed their involvement in various processes including cell proliferation and cell migration (Fig. 7d and Figure S6-a). We thus evaluated the contribution of SET7 and ERRα in these two processes. We found that cell proliferation was not influenced by these factors (Figure S6-b and^26^). In contrast, using wound healing experiments in MDA-MB-231 cells, we noted that cell migration highly depended on ERRα and SET7 with no additive effect (Fig. 7e).

## Discussion

Our approach identified twenty-four TRs as robust quantitative predictors of the expression of ERRα-activated target genes in association with *ESRRA*. The selected TRs include specific DNA-binding TFs, histone modifiers, RNA modifiers as well as TF-binding co-regulators. Although detected across a set of various BC cells, activation of the expression of ERRα targets by four TRs was confirmed in different cell models such as MDA-MB-231 cells. Moreover we showed that SET7, encoded by the *SETD7* gene, physically interacts with ERRα, and that the transcriptional effects of SET7 depend on the presence of ERRα specifically on their common target genes. At the cellular level, these two factors favor cell migration in a non-additive way further supporting their coordinated activity.

### Performances of the sPLS modeling method

The PLS regression approaches the problem of multicollinearity between predictors by feature extraction. In PLS regression an orthogonal basis of latent variables (linear combinations of predictors), not directly observed or measured, is constructed in such a way that they are maximally correlated with the response variable. PLS regression is therefore mostly a compression approach. Modeling in genomics always faces the problem of a high number of predictors (genes) collected for a small number of samples. Sparsity-based approaches are then necessary to propose biologically usable results. Sparse PLS methods have already shown good performance for regression with a continuous quantitative response^37,38^. In this work we used an adaptive sPLS approach including the choice of parameters by cross-validation and an improvement of the feature selection process^39^.

Although we analyzed only genes identified as ERRα–activated targets, only 25% of them included ESRRA with a positive coefficient in good quality models. This result may arise from the lack of robustness of these genes as ERRα–activated targets in all BC cell types. Indeed, they were first identified in MDA-MB-231 cells but only partially confirmed as ERRα–activated in other BC cells such as MCF-7 or SKBr3 cells (Figure S4–S5). Another reason could come from the fact that the expression data of several of the 318 TRs may contain redundant information with *ESRRA*. The main limit of this study aiming at uncovering actors of transcriptional regulation is the unique use of static mRNA expression data, taking into account neither the post-translational modifications of TRs nor the dynamics of gene expression regulation. In addition, the modeling approach hypothesized multiplicative contributions of TRs on gene expression (sum of logs), which is classically used for DNA-binding TF cooperation^40,41^. The establishment of expression models across several cell types analyzed in a population approach is also a limit. Indeed, it makes emerge features from the variability of expression across cell populations, but we cannot prove that the models are valid for each of the cell types.

Our in vivo analysis in MDA-MB-231 cells confirmed the involvement of the three selected TRs in expression activation of ERRα-activated genes. It did not highlight any false positive genes from sPLS models but identified some false negatives. Several reasons may account for this. First, as stated above our results emerging from various BC cells may be not relevant for all of BC cell types. Some ERRα-activated genes may thus involve an ERRα partner TR in MDA-MB-231 cells but not in other BC cells so their expression model across cells does not include this TR. Second, some TRs may be actual ERRα partners for some genes undetected by our method due to information redundancy across TRs in the expression data.

Nevertheless, this statistical modeling approach is particularly suited to investigate the combinatorial control of gene expression due to its variable selection feature. Although we could not reveal the dynamic complexity of the transcription process, we could identify TR combinations as determinants of the expression of ERRα–activated target genes.

### DDX21, MYBBP1A, and NFKB1 as ERRα potential partners

Our bio-computing approach allowed to identify a number of novel ERRα-associated TRs as potential coactivators. ERRα is involved into several pathophysiological processes such as the regulation of energy metabolism, including in breast cancers^20,22,42,43^. These regulations are exerted through the activation of “metabolic” target genes and highly depend on interaction with members of the PGC-1 (*PPARGC1A* and *PPARGC1B*) family of transcriptional co-activators. Our current approach did not identify members of this family as potential ERRα partners. Indeed, PGC-1 factors were not among the most informative TRs used for modeling, due to their low expression in the studied BC cells (0.14 and 0.60 q75-normalized TPM in CCLE data, and 0.73 and 0.84 q75-normalized FPKM in GSE58135 data). Similarly, other described ERRα co-activators such as those of the SRC family^44,45^ could not be identified because the *NCOA1*, *NCOA2*, and *NCOA3* genes encoding them were not part of our short list of TRs due to insufficiently informative expression content across BC cells compared to the other TRs. Nevertheless, we performed an additional computation after adding all these factors to our previous TR list (Figure S7). With the exception of *PPARGC1B* (again, weakly expressed in the studied BC cells), none of these factors were suggested as potential ERRα partner. This suggests that regulation of gene expression by ERRα in MDA-MB-231 cells involves different TRs than those already identified. Furthermore, the above-mentioned metabolic genes were not found as ERRα-activated targets in MDA-MB-231 cells^30^ on which the current study is based, although binding of the ERRα protein is clearly detected on the promoters of these genes by ChIP-seq approach (Figure S1). Altogether, this suggests that at least two transcriptional programs can be regulated by ERRα: a “metabolic” one which requires PGC-1s and a “migratory” one, involving other TRs.

The identified TRs were predicted to display a number of target genes in common with ERRα, and our independent RT-qPCR experiments verified this hypothesis for three of them. However, the exact mechanism through which these common effects are exerted is currently unknown. The RNA helicase DDX21 is a key regulator of ribosome biogenesis^46^, participates in transcription regulation as a cofactor^47,48^ and is involved in the progression of various cancers including breast cancers^47,49^. The MYB-binding protein 1A, first identified for its binding to c-MYB, is a nucleolar protein that may translocate to the nucleoplasm and enhances p53 activation^50,51^. Together with DDX21, MYBBP1A is part of the B-WICH complex that remodels chromatin and recruits histone acetyltransferases for transcription activation of rRNA genes^52^. The NF-κB complex associates five proteins: p50 and p52 from their precursor p105 and p100, RelA/p65, RelB and c-Rel encoded by *NFKB1*, *NFKB2*, *RELA*, *RELB*, and *REL* genes respectively. They form homo- or hetero-dimers among which p50/RelA is part of the canonical NF-κB signaling pathway. Its involvement in innate and adaptive immunity is well known as well as in cancer progression due to constitutive activation by multiple oncogenic signaling pathways^53,54^. Only *NFKB1* was present in our shortlist of 318 TRs making it the unique component of the NF-κB complex in our analysis.

### SET7 as an ERRα co-activator involved in BC cell migration.

GO analysis performed on targets regulated by ERRα in MDA-MB-231 cells showed a strong enrichment in terms related to cellular migration, but not to those related to energy metabolism^26^, altogether indicating that the repertoire of targets activated by ERRα is dictated by the action of co-regulators rather than by direct DNA binding. Consistently, we identified SET7 as a TR involved in the co-regulation of cell migration by ERRα. We found that these proteins interact together and co-localize in cell nucleus. Unbiased RNA-seq showed that SET7 activates the expression of a small number of ERRα-activated target genes in MDA-MB-231 cells. Our results of siRNA experiments showed that there was no synergic effect of ERRα and SET7 on gene expression, suggesting that both factors act in the same pathway. Furthermore, rescue experiments indicated that the effects of SET7 were dependent on the presence of ERRα, specifically on SET7- ERRα co-targets, but not on SET7-specific targets (*i.e.* not regulated by ERRα). Although the precise molecular mechanism through which ERRα and SET7 co-activate target genes is currently undetermined, our GO analysis strongly suggests that these targets are mainly involved in the regulation of cell migration. Functional analysis shows that this phenomenon indeed requires both ERRα and SET7 in a non-additive manner. Interestingly, transcriptional co-regulation by ERRα and SET7 was confirmed in the migratory SKBr3 and HeLa cells, but not in the non-migratory MCF7 cells. Consistent with our data, high SET7 expression correlates with cancer aggressiveness^35,36^, in a similar manner to that observed for ERRα^20,22^. These results are in line with studies showing SET7 as an activator of other nuclear receptors such as AR or FXR^55,56^.

In summary, we here described an in silico method that allows to propose co-regulators driving a transcriptional program dedicated to a particular phenotype. This approach was here operated using publicly available expression datasets from cells in culture. While our current conclusions may be limited to these in vitro systems, the use of expression datasets from in vivo tissues may be envisioned to question numerous phenotypes.

## Materials and methods

All the experiments were performed in accordance with the relevant regulations or guidelines.

### Identification of direct ERRα-activated genes

Genes regulated by ERRα in MDA-MB-231 cells have been previously identified by RNA-seq in a previous study of our team^26,30^ with data retrievable in GEO (GSE49110). ChIP-seq data were specifically generated for this study and performed by the NGS IGFL platform. Two ChIP replicates using an ERRα antibody along with two input samples were obtained (see below) and libraries prepared with the ACCEL-NGS 2S Plus DNA Library Kit (Swift Biosciences) following manufacturer’s instructions. ChIP DNA libraries were then sequenced in paired-ended mode (2 × 81 bp) using the Illumina Next Seq 500 sequencer yielding to 29 M and 10 M reads for ERRα and input samples, respectively. The analysis pipeline included adapter trimming and quality control with TrimGalore! (v0.6.4) based on Cutadapt and FastQC, hg38 genome alignment with Bowtie2 and identification of peaks with MACS2 (v2.2.6) (Figure S1-a-b). Identified peaks were further annotated using the ChIPseeker R package for their genomic location and their biological function (Figure S1-c-d). The presence of a consensus ERR-response element (ERRE) was checked in the ± 250 bp region around the peak summit using the FIMO tool of the MEME suite and the Jaspar matrix MA0592.3. Sequences showing motif score with *p*-value < 10^–4^ were taken as positive for ERRE. Direct ERRα-activated targets were those genes with reduced expression upon siERRα and that showed a ChIP-seq peak in the ± 100 kb region from TSS containing an ERRE.

### Public expression datasets

Public RNA-seq data from BC cell lines were used to find potential TRs cooperating with or activating ERRα in this cancer context. One dataset derived from the Cancer Cell Line Encyclopedia of the Broad Institute (CCLE)^57^. We selected data obtained in 51 BC cell lines. A second dataset came from the Gene Expression Omnibus (GEO) database (accession number GSE58135). In this study, RNA-seq data were obtained in 28 BC cell lines^58^. For each dataset, expression data in TPM or FPKM were further submitted to upper quartile normalization between samples after removal of genes not expressed in all of the samples. For the GSE58135 dataset, only expression values with status OK were taken into account, other ones (LOWDATA or FAIL status) were replaced by NA. Details of cells and expression data are given in Figure S7.

### TR collection for modeling

The human TRs were collected in 02/2019 from several public databases containing both DNA-binding TFs and non-DNA-binding TRs: HumanTFDB3.0 (http://bioinfo.life.hust.edu.cn/HumanTFDB#!/), as well as dbEM (https://webs.iiitd.edu.in/raghava/dbem/) and Epifactors (http://epifactors.autosome.ru/) for epigenetic modifiers. Merging all of the collected identifiers led to 2175 unique human TRs. A pre-selection of these TRs was based on 1-expression values (mean and SD > 0.1 after upper quartile normalization), and 2-principal component analysis (FactoMineR R package) across cell lines using log2 expression values of TRs taken as variables. For each TR, scores giving correlation with each of the first five principal axes were analyzed. TRs with absolute value of score (= correlation with the principal axis) higher than 0.5 for at least one axis were considered as those best explaining the variability of TR expression across samples. Then after inclusion of a few other TRs of potential interest, the TRs identified in both datasets were pre-selected for model computation.

### Sparse PLS models

Using the expression of several hundred genes as explicative variables in a regression model has major limits related to the number and the collinearity of these variables. To tackle these limits, we used an adaptive sparse partial least squares (sPLS) regression method for univariate responses^39^. The procedure combines compression, building a limited number of orthogonal latent components (1 to 5), and variable selection. It is implemented in the plsgenomics R package.

Univariate sPLS models were computed for one gene at a time (Figure S8-a). The contribution of each TR to gene expression was taken as the number of genes including the TR in the computed model, allowing identification of the most frequent ones (Figure S8-b). Gene selection for expression modeling was based on expression level (median value > 0 and no missing value) and expression variability across samples (SD > 0.01). For each gene, sPLS modeling was applied on log2 expression data previously upper-quartile normalized in each sample. Expression data were further standardized in the model computation steps. The optimal number of latent components and the optimal lambda parameter were first determined using the spls.cv function that implemented a K-fold cross-validation method: 1 to 5 PLS components and K = 10 or 7 according to the number of samples in the dataset. Then the model was computed with the spls function and these optimal parameter values. The residual variance and the R-squared determination coefficient were used to estimate the quality of the model. For the ERRα-activated genes, the computation was replicated 10 times. In addition, 100 sets of random genes were generated. For each dataset and each set of random genes, the same number of adequately expressed genes as ERRα targets was submitted to modeling. Models were computed one time for each random gene set.

### Computational strategy for TR selection

In each dataset, TRs were selected over the whole set of ERRα-activated genes. The TR selection procedure comprised two main steps: 1. select genes showing a suitable model (R-squared > 0.6) that includes less than half the total number of TRs and shows a positive coefficient for *ESRRA*; and 2. for each TR, compute the proportion of these genes with sPLS regression coefficient > 0, then the mean proportion over ten replicates for ERRα-activated genes; then compute the fraction of random gene sets for which the proportion of genes with reliable sPLS model and positive coefficient (but no criterion on the *ESRRA* coefficient value) is greater than the proportion obtained for ERRα-activated genes. This fraction value gives the *p*-value of non-specificity of the TR, taken as the significance value of the TR for the genes of interest. Lastly, only significant TRs (*p*-value < 0.05) identified for > 20% of ERRα-activated genes with a positive model coefficient for the TR were selected.

### Identification of ERRα- and SET7-activated genes

Genes activated by SET7 were identified from a RNA-seq experiment performed in MDA-MB-231 cells treated by a siRNA targeting SET7. Two siRNAs targeting SET7 (Table S5) were compared to a control siRNA. RNA-seq was done on triplicate samples and libraries build using the mRNA-Seq Library Prep kit of Lexogen following the instructions for 5500 SOLiD and including a conversion step to SOLiD 5500 W. Sequencing was performed by NGS IGFL platform with SOLiD 5500 W System (Life Technologies). Sequences were aligned on the human genome (hg19 version) in color-space using the Lifescope dedicated software. Read counts were determined using HTSeq v0.6.1. Differentially expressed genes were identified with DESeq2 R package, using an adjusted *p*-value < 0.05 and fold change threshold of 1.5 and 0.75 for over- and under-expressed genes respectively. Genes showing significantly modified expression with both siSET7 were considered as SET7-modulated genes.

These genes were gathered together with the previously identified ERRα-modulated genes for hierarchical clustering (Cluster 3.0) and heatmap representation (Java TreeView 1.1.6r4). Clustering used the euclidian distance and the average linkage method applied to log2 mean fold changes over two siRNAs for ERRα or SET7. Genes repressed by siERRα and siSET7 were selected and further tested for their association to an ERRα ChIP-seq peak showing an ERRE at summit location.

### Cell culture

All cells originated from ATCC and were cultured in DMEM supplemented with 10% FCS, 10 U/ml penicillin and 10 µg/ml streptomycin. For siRNA transient transfection, 3.10^5^ cells per ml were seeded in 6-well plate and 25 pmol/ml of siRNAs (Eurogentec) were transfected with INTERFERin (Polyplus Transfection) according to the manufacturer’s instructions. Plasmid pCDNA-HA3-SET7 (a generous gift of I. Talianidis) was used to introduce mutations in the siRNA recognition site. This construct was then transferred into pSG-Puro plasmid. Stable MDA-MB231 transfectants were selected for their puromycin resistance and maintained as populations. For proliferation assays, 10^4^ siRNA-transfected cells were seeded in 96 well plates. Cell viability was determined 48 h after transfection using CellTiterGlo kit (Promega) under the manufacturer’s recommendations. For migration analysis, cells (5 × 10^5^) were seeded on 6-well plates (Falcon) and grown to 100% confluency for 48 h. Cell layers were scratched with a plastic pipette and washed twice with PBS. Images of wounded monolayers were acquired for 0 to 12 h using a Timelapse Axiovert100M microscope. Individual cell tracking was analysed (in terms of velocity, total distance, Euclidian distance). Cell-free spaces were quantified with ImageJ software.

### RNA expression analysis

Total RNAs were extracted by the guanidinium thiocyanate/phenol/chloroform method. 1 µg of RNA was converted to first strand cDNA using the RevertAid kit (ThermoScientific). Real time PCRs were performed in 96 well plates using the IQ SYBR Green Supermix (BioRad). Data were quantified by ΔΔ-Ct method and normalized to 36b4 expression. Significance was evaluated using t-test comparing specific siRNA to control ones. Primer sequences used for these experiments are shown on Table S5.

### Protein analysis

For Western blot analyses, cells were lysed in RIPA buffer supplemented with protease inhibitor cocktail (Sigma-Aldrich). Proteins (25–50 µg) were resolved in 8% SDS-PAGE, blotted onto PVDF membrane (GE-Healthcare) and probed with specific antibodies after saturation. The antibodies used in this study were: hsp90 (API-SPA-830, Enzo Life Sciences), ERRα (GTX108166, Gentex), Set7 (#2813, Cell Signaling Technology). For co-immunoprecipitation assays, cells were harvested in Phosphate Buffered Saline (PBS) and pellets were resuspended in lysis buffer (50 mM Tris pH7.5, 150 mM NaCl, 1 mM EDTA, 1% Triton X-100, 8% glycerol) supplemented with protease inhibitor cocktail (Sigma-Aldrich). 800 µg to 1 mg of proteins were pre-cleared for 1 h on Sepharose-protein A (GE-Healthcare) with binding buffer (20 mM Tris pH7.5, 150 mM NaCl, 1 mM EDTA, 8% glycerol) and 2 µg of antibodies were added for 3–4 h at 4 °C with rotation (ERRα, PP-H5844-00, R&D; SET7, #2813, Cell Signaling Technologies). Beads were then added to the extract and incubated for 1 h, washed 3 times with wash buffer (20 mM Tris pH7.4, 150 mM NaCl, 0.1% Triton X-100, 1 mM EDTA) and finally resuspended in Laemmli buffer for immunoblotting analysis. 10% of whole cell lysate were analysed as input fraction.

For proximity ligation assays cells cultured on coverslips were fixed with 2% paraformaldehyde (Merck) for 10–20 min at room temperature, washed with PBS, and analyzed with the Duolink PLA kit (O-link; Bioscience) according to the recommendations provided by the manufacturer using anti-SET7 or anti-ERRα antibodies. Samples were Dapi-counterstained. Images were acquired using a Zeiss AxioImager microscope.

### Chromatin immunoprecipitation

10 × 10^6^ cells were cross-linked with 1% formaldehyde and quenched for 5 min in 1 M Glycine. After centrifugation, cell pellets were resuspended in lysis buffer (1% SDS, 50 mM Tris–HCl pH8, 10 mM EDTA). Sonication was performed with Ultrasonicator (Covaris). Lysates from 2.5 × 10^6^ cells were processed with the iDEAL ChIP kit (Diagenode) according to the manufacturer’s recommendations using 5 μg of antibody (ERRα: GTX108166, Genetex; IgG provided in the Diagenode kit). Quantitative PCRs were performed using 2 μl of DNA in duplicate and enrichment was calculated related to input. Primer sequences and siRNAs used for these experiments are shown on Table S5.

## Supplementary Information

Supplementary Information 1.

Supplementary Information 2.

Supplementary Information 3.

Supplementary Information 4.

Supplementary Information 5.

Supplementary Information 6.

## Data Availability

RNA-seq and ChIP-seq data obtained in MDA-MB-231 cells can be retrieved from the Gene Expression Omnibus portal, accession number GSE49110 for RNA-seq data using siRNA against ERRα, GSE163017 for RNA-seq data using siRNA against SET7, and GSE163166 for ChIP-seq data targeting ERRα. ChIP-seq data can be visualized using http://genome.ucsc.edu/s/cerutti/ERRA_ChIPseq_hg38_public.
