# Supplementary material for: Computational identification of new potential transcriptional partners of ERRα in breast cancer cells: specific partners for specific targets
Source: Sci Rep. 2022 Mar 9;12:3826. doi: 10.1038/s41598-022-07744-w (PMC8907200; doi:10.1038/s41598-022-07744-w)
Supplement: Supplementary file 5 — Supplementary Information 5. [file 41598_2022_7744_MOESM5_ESM.docx]

**Supplementary Table S5: Oligonucleotides and siRNAs used in this study**

**Oligonucleotides for expression studies**

| 36b4 | GTCACTGTGCCAGCCCAGAA | TCAATGGTGCCCCTGGAGAT |
| --- | --- | --- |
| ESRRA | CAAGCGCCTCTGCCTGGTCT | ACTCGATGCTCCCCTGGATG |
| MYBBP1A | TGCCCTCCAAGCTCAAGAAG | CTTCTTCACAGAGGAGGCGG |
| NFKB1 | GCACCCTGACCTTGCCTATT | CATCTCCTTGGTCTGCTGCA |
| DDX21 | TTCTCAAAGGCCGAGGAGTG | GTCTTCCCAGTTCCTGTCCG |
| SETD7 | GGGAACTTTGTTCACGGAGA | ACATACGTGCCCTGGAGAAC |
| CLUH | GACCCATCCGATGCCTTCAA | GGTCCATCTCCAAGCCCTTC |
| CLPB | ACACCCTTGGATTATGCCCG | TGGCCAATGATGTGCTCCTT |
| NDUFAF4 | GACACCCCTCTACCAACAGC | AGAAACGACAGCAGCTTTTCA |
| SINHCAF | CCGAAGTATAGAGGGCTGCTG | ACAAGCAGGACACAGGCATT |
| NOP2 | TCAATGTGGATGAGGAACCA | ATATCCTGGATCCGCTTGTG |
| CHCHD10 | GTGACCTGTCCCTGTGTGAG | GTCTGCACCGACCTCTTCAG |
| GOT2 | ATCATTGGCATGCGGACTCA | AACTCCTTGATCAGCCGCTC |
| NUDT19 | TGGCTGCCGATCATCTTGTT | ACTGCTTGCCTTCCTTCATGA |
| TMEM120B | AATGTGAACGTGACCCTCCT | AGACACCCACCAGCCTTTAA |
| POP1 | ACAGTCAAGAGCCACAGAGC | ACACATTCCAGAAAGCGCCT |
| BDH1 | GCATTCAAAAGGCTTCCTTG | TCCACCACTTTCTCCACCTC |
| CELF1 | CCAAGGACCTGGTCTGAAAA | AACAAAACAGCACCCTTTGC |
| ESM1 | TGTGACAGCAGTGAGTGCAA | TGAGACTGTGCGGTAGCAAG |
| FAM155B | AGCAATACTGCCTGGAGGTG | CACAGCACTTGGTTTCCAGA |
| KLHL18 | GTGCACTTCTCCGTGTCTGA | CGAGGCTGCTAAGACAATCC |
| LMNB1 | CCTCAGCCACTGGAAATGTT | CTCCCATTGGTTGATCCTGT |
| NFATc2 | AAAACAACATGAGGGCAACC | CGCGTGTTCTTTCTTCCAAT |
| PHACTR1 | GCTCCAACCGTCAGACATCA | GGACTGGTTTGGCTGAAGGA |
| PHLDB2 | GACTCAGGGAGCAGGAAATG | AAGCGACTGTCAGGCTTTGT |
| PPM1E | GCAGGAAAATGGAGGACAAA | GGAGGTGAATGGAGGCATAA |
| RAI14 | TACATTATGCAGCGGCTCAG | ACAGCAAGAAGCAGCGGTAT |
| SAMD12 | AGAAGGTGCCTGACCAGAAA | ACATCCTGCTGGGTCCATAG |
| SAMD4A | ACCAGTGGATTCGTCAGCTC | GTTGGCAAACCACAGGTTCT |
| SFTA1P | AGCCGAATACAGTTGCCAAG | GGTGGTCTGCCATCTCACTT |
| SNCAIP | CGCAAAACGAAGACAGATCA | CTGATGTTTCAGTGGCGAAA |
| BRCA1 | TAAGCCGCAACTGGAAGAGT | CCGGATGACGTAAAAGGAAA |
| USP16 | AACCCCTAAACCCGAACATC | GGCATTATGGGAAGTGGCTA |

**Oligonucleotides for ChIP**

| CLUH | GGGGCGACTGTGAAGGTG | CGGTTGTATCTCGCTGGGG |
| --- | --- | --- |
| CLPB | AACCTGTGTACACCCTAGCA | TGTGGCTTGCAGTGGATTGA |
| NDUFAF4 | GCCACCAAAGAGTTAGCCGA | CACAAAAGCAGGAAAGGGCA |
| SINHCAF ERRE1 | ACCAAACACTGAGTCACGCA | TGTTTCTGGTGGAGTTTCGGA |
| SINHCAF ERRE2 | CTTCCTGAGCAGCTTCCTCC | GCGAAAGGTATGCGTGACTC |
| NOP2 | CCGTTCCATGTACTTCACTGGT | CGCTTAATGGCTCTGTGACC |
| CHCHD10 ERRE1 | GCCCACACTTCCCTAACCC | GCCGTCTCTAAGGTCGCC |
| CHCHD10 ERRE2 | GACCTAGCCACAAAGCCACA | GGATTTCCTGAGCCCCTGTT |
| CHCHD10 ERRE3 | CCCCGGAGAGATGGACGA | GGGTTAGGGAAGTGTGGGC |
| GOT2 | AAGGTCAGGAGAATGCCGC | GAGCGCGAGAGGAAGGTTTC |
| NUDT19 | GGTTCACGTGTGCTTGCAAA | CCAGGCCTGATGACATCCAC |
| TMEM120B | ACCTCACAGTGATGCAAGGG | AGCCTGTCTCCCTGATGTCT |
| POP1 | CGGTCTTGGCATCTTGGAGT | CAGGGCAAAGGATCGAGGTT |
| BDH1 | TCAGAACAAAGGGGCCACTC | CCAAAGAGGTCAGGTCATGCA |
| CETN3 | GCTTCACTGTTCCAATAAAGTCA | GTCGCACACTGATGAGTTCC |
| DARS | GGGAATTCGCGGTAGTTTCG | ATGGAGCAGAAGTCGCAGTT |
| intergenic | AGTCCATTTTCACACTGCTGA | TGCCTTTCACCTTCCAATTGTG |
| CELF1 | GAATCCTATGTTGATGATGCCCA | GAATGGGAGTGTCAGTGGGG |
| ESM1 | TGCTCAGAAAGGTGAAGTAGC | TCCCACTCCCAGACTTACCT |
| FAM155B ERRE1 | AAACTTCTTGACCCTCGGCG | ACCCTTTGACCTTGCTGTTCT |
| FAM155B ERRE2 | CCAAGGATAGGAGAGGGGCA | ACAGTGGATTGACAGGCCTG |
| KLHL18 | GCACATGCGAAGTCAAGGTC | TAGTTGAATCTCCTCGGCGC |
| LMNB1 | GCAGAGGCCAAGCAAAGTTC | TGCTGGTTGTTCAACATGCC |
| NFATc2 ERRE1 | GCGTGGGTTCGTGTGTTTT | GGACTCAGTGGTGCCTTCC |
| NFATc2 ERRE2 | TTACCACCACGTTGCCATGA | GTTTTGAGACCCCACTTCGG |
| PHACTR1 | CGTCAGTGATAGCCTTGGGG | TTGTGGTTGAATGTGCAGGG |
| PHLDB2 ERRE1 | AAAGGTGACGTGACTTGCCC | TGTCCCTCACTCTCTGCCTT |
| PHLDB2 ERRE2 | ACAGAGTGATCCAGGACGTC | TGGAAAGCTGAATTGACCTCTGA |
| PPM1E | TGAGTCCTTGTTGCATCCCT | CCACGCCGGCCAATTATTC |
| RAI14 ERRE1 | GCCCGGTCTCCCAAACTAAG | ACTTGAAAATGAGACCCTGGTG |
| RAI14 ERRE2 | GGTGTGCTCTGACCTTGACG | GGGGTGCACACATTATTAGGC |
| RAI14 ERRE3 | TCAGACTGAGTTGTGCGTGG | GTGAGGAAAGCGGAGGATGT |
| SAMD12 ERRE1 | CCCAGTCTAAGGCATTCCCC | TGAAAGGCATTTATCCGCTCA |
| SAMD12 ERRE2 | GTGAGAGGAGTGCAGCCAAT | AGCAGATGACCTTGGCCTTC |
| SAMD4A | CATTCCCAAGGACAAGCAGC | GTGAATTTCTTCCTGCGGCC |
| SFTA1P ERRE1 | ATCCCTGTTGCTCATCCTGC | TGACCTCCAACTTGCTGACT |
| SFTA1P ERRE2 | GAGTCACGACCTTCAGCCAA | CACCCAGTGTTGACTTCGGA |
| SNCAIP | TGCAAAATGGTGGCTGTTCC | ACAGGTGAAATGGCTTGGGT |

**siRNAs**

| ERRα | GAAUGCACUGGUGUCUCAUCAGCUG | CAGCAGAUGAGACACCAGUGCAUUC |
| --- | --- | --- |
| MYBBP1A | CCUAUUCUCAGAUGAGAAU | AUUCUCAUCUGAGAAUAGG |
| NFKB1 | CCGAAGCAAUUGAAGUGAU | AUCACUUCAAUUGCUUCGG |
| DDX21 | CCUUCCUAUUUCCUAUACA | UGUAUAGGAAAUAGGAAGG |
| SETD7 | GCCUUGUAGGAGAAGUAAA | UUUACUUCUCCUACAAGGC |
