# Supplementary material for: Computational identification of new potential transcriptional partners of ERRα in breast cancer cells: specific partners for specific targets
Source: Sci Rep. 2022 Mar 9;12:3826. doi: 10.1038/s41598-022-07744-w (PMC8907200; doi:10.1038/s41598-022-07744-w)
Supplement: Supplementary file 6 — Supplementary Information 6. [file 41598_2022_7744_MOESM6_ESM.pdf]

Computational identification of new potential transcriptional partners of ERRα in breast cancer cells: specific partners for specific targets. C Cerutti, L Zhang, V Tribollet, JR Shi, R Brillet, B Gillet, S Hughes, C Forcet, TL Shi, JM Vanacker.

Figure S1

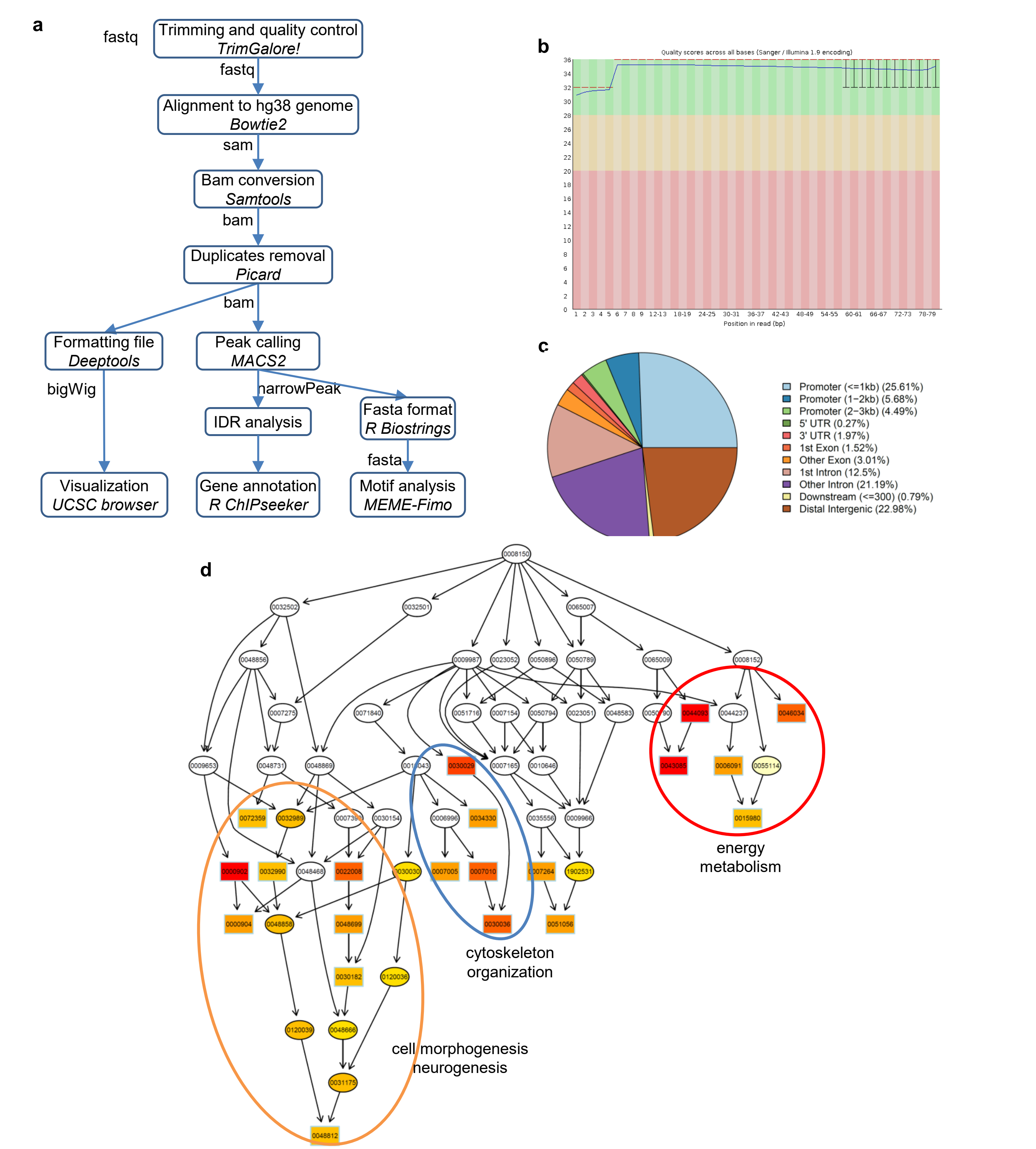

Figure S1. Analysis of ChIP-seq data for ERRα in MDA-MB-231 cells. **a.** Flowchart of the analysis procedure. **b.** Example of quality score of sequenced reads obtained with FastQC for one ChIP ERRα fastq file (sample S4, R1 file) after trimming. All samples gave as good results. **c.** Example of genomic annotation of peaks obtained for one sample with R ChIPseeker package (using transcript option). Most of the peaks (82%) were distributed between transcriptional start site (TSS) regions (≤ 1kb), introns and distal intergenic regions, suggesting various DNA-binding modes for ERRα. **d.** Tree representation of GO terms (Biological Process category) enriched in genes associated to ChIP-seq peaks. Numerical GO IDs are shown. Three main families were over-represented: cell morphogenesis, energy metabolism and cytoskeleton organization.

Figure S2

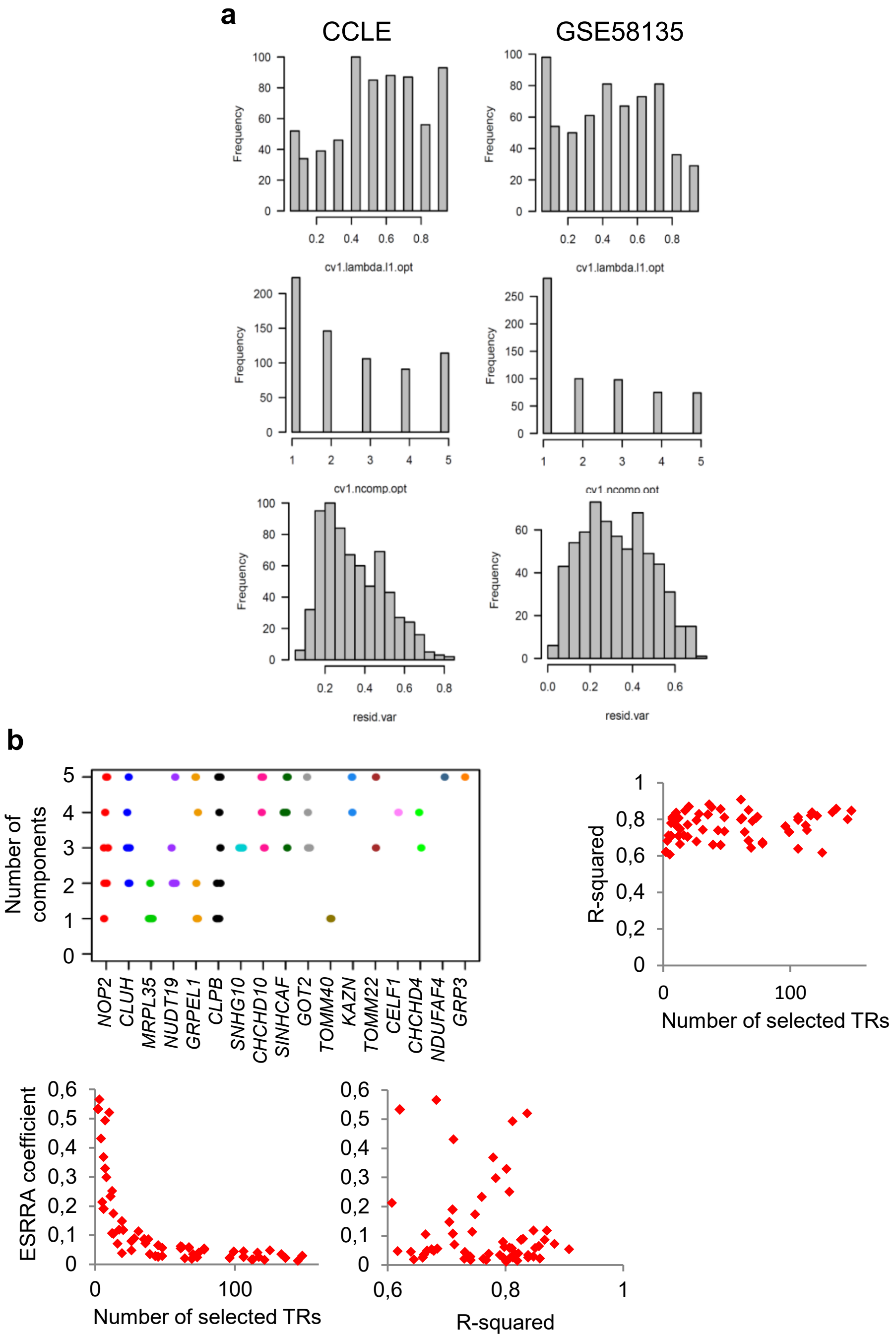

Figure S2. Features of computed expression models.

a. Frequency histograms of model features across all ERRα-activated genes and all model replications (10 times) using CCLE and GSE58135 data (680 models for CCLE and 630 for GSE58135): lambda coefficient, number of latent components and residual variance.

b. Results of good quality models with ESRRA coefficient > 0 obtained for 17 ERRα-activated genes using CCLE data (98 models). Top left panel: Number of latent components used in suitable expression models obtained for the 17 ERRα-activated genes across the 10 replicates. Right panel: lack of relationship between the number of TRs in the model and the R-squared value of the model. Bottom panels: consistent inverse relationship between the ESRRA coefficient and the number of TRs in the model, but lack of association with the R-squared value.

Figure S3

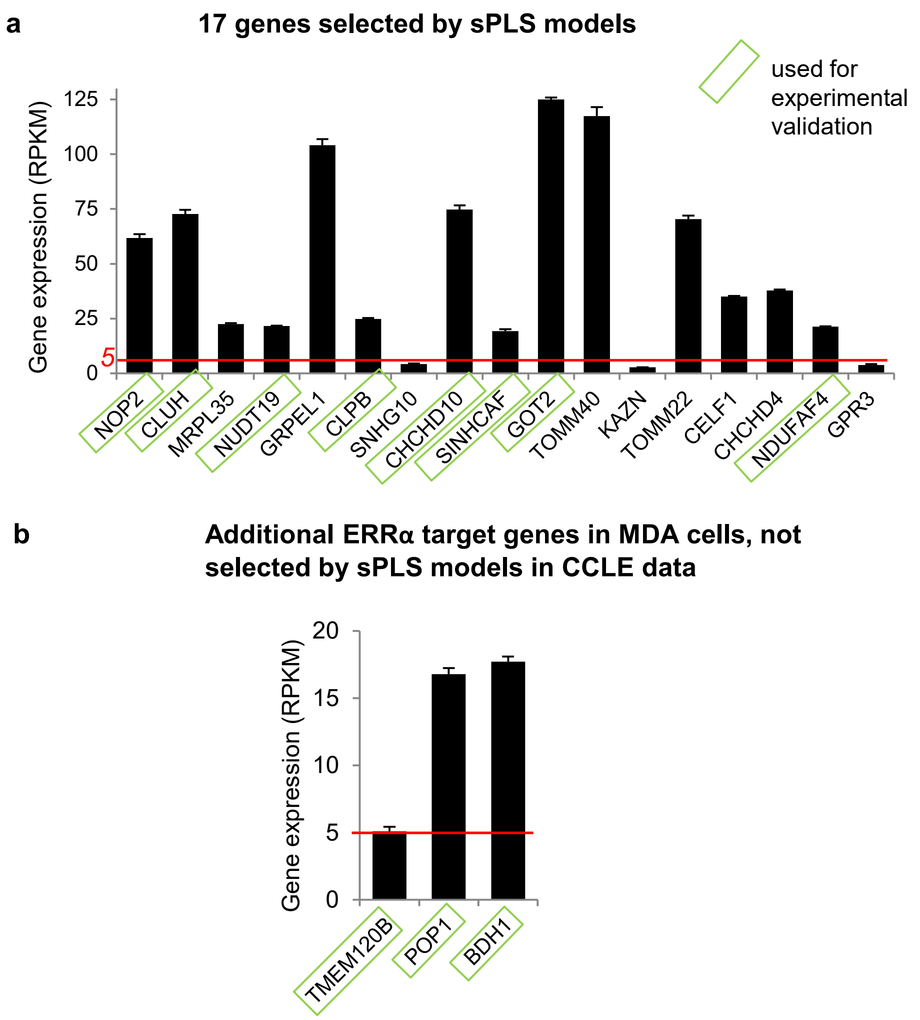

Figure S3. Expression of ERRα-activated genes in MDA-MB-231 cells.
RNA-seq expression data were obtained in duplicate in MDA-MB-231 cells treated by a control siRNA. Data are mean ± sem. **a.** Expression of the 17 ERRα-activated genes identified from sPLS models computed in BC cells. The red line located at 5 RPKM indicates the expression threshold. Genes outlined in green are those used to test the effect of the selected TRs in MDA-MB-231 cells. **b.** Expression of 3 ERRα-activated genes used as negative controls for the selected TRs.

Figure S4

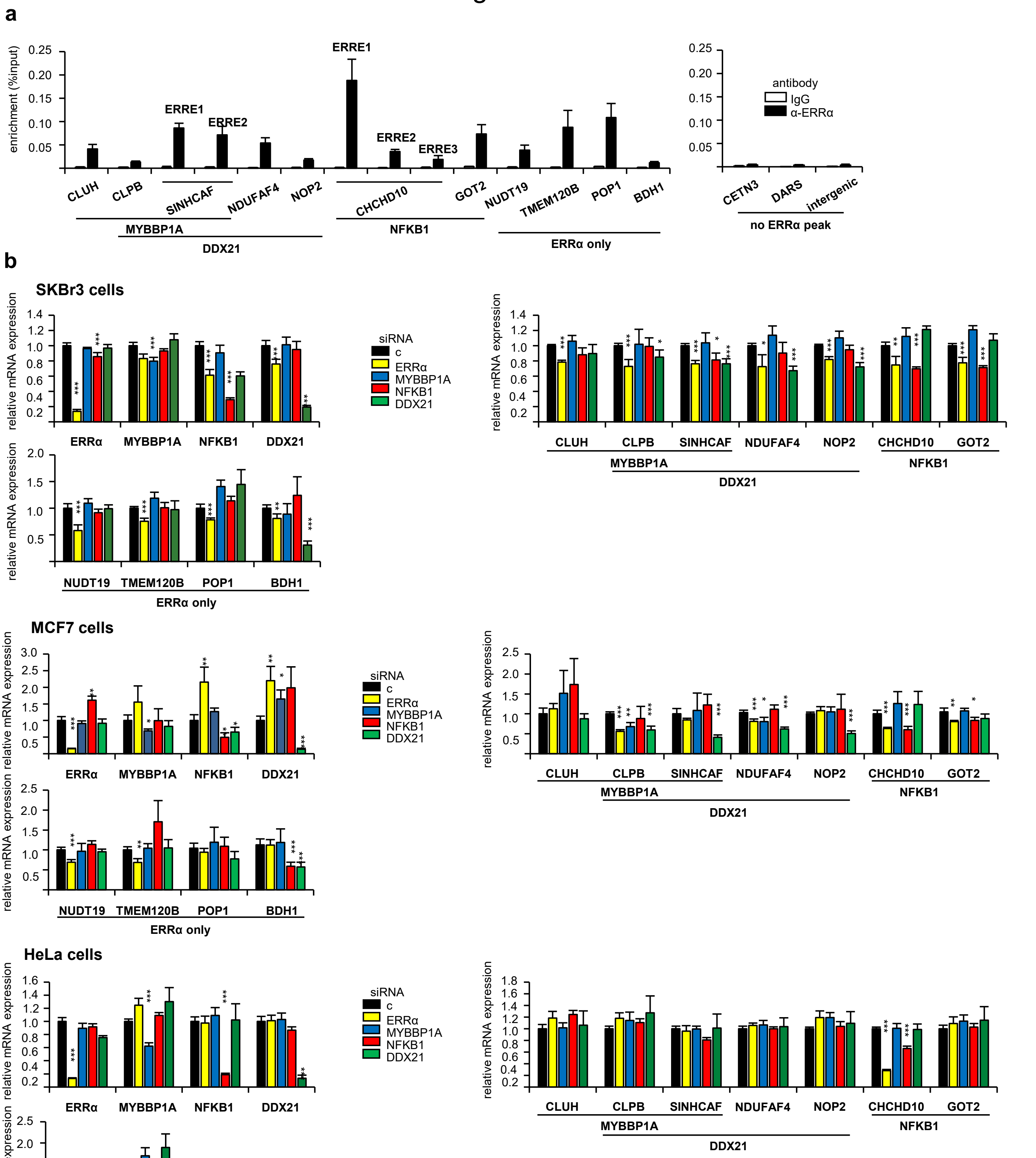

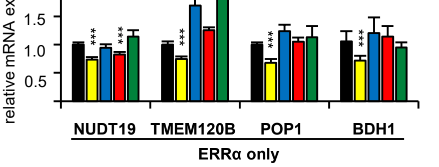

Figure S4. Validation of ERRα target genes in MDA-MB-231 cells and regulation of their expression by ERRα and suspected co-regulators in cancer cells in SKBr3, MCF7 and HeLa cells. a. Binding of ERRα to genomic sequences identified by ChIP-Seq was confirmed by ChIP-qPCR experiments performed on independent samples. Percent enrichments after IP with anti-ERRα antibody or IgG were measured by qPCR amplifying a region encompassing the identified ERRα response elements (ERREs). CETN3, DARS and intergenic represent genomic regions where no ERRα peak was detected in ChIP-Seq experiments and were used as negative controls. Values are expressed relative to input. Bars represent mean +/- sem of two independent experiments performed in duplicate. **b.** Same as **Figure 5** with experiments performed in SKBr3, MCF7 or HeLa cells. As evaluated by t-test, variations are not significant unless indicated by ***: p<0.001, **: p<0.01, *: p<0.05 vs si-c.

Figure S5

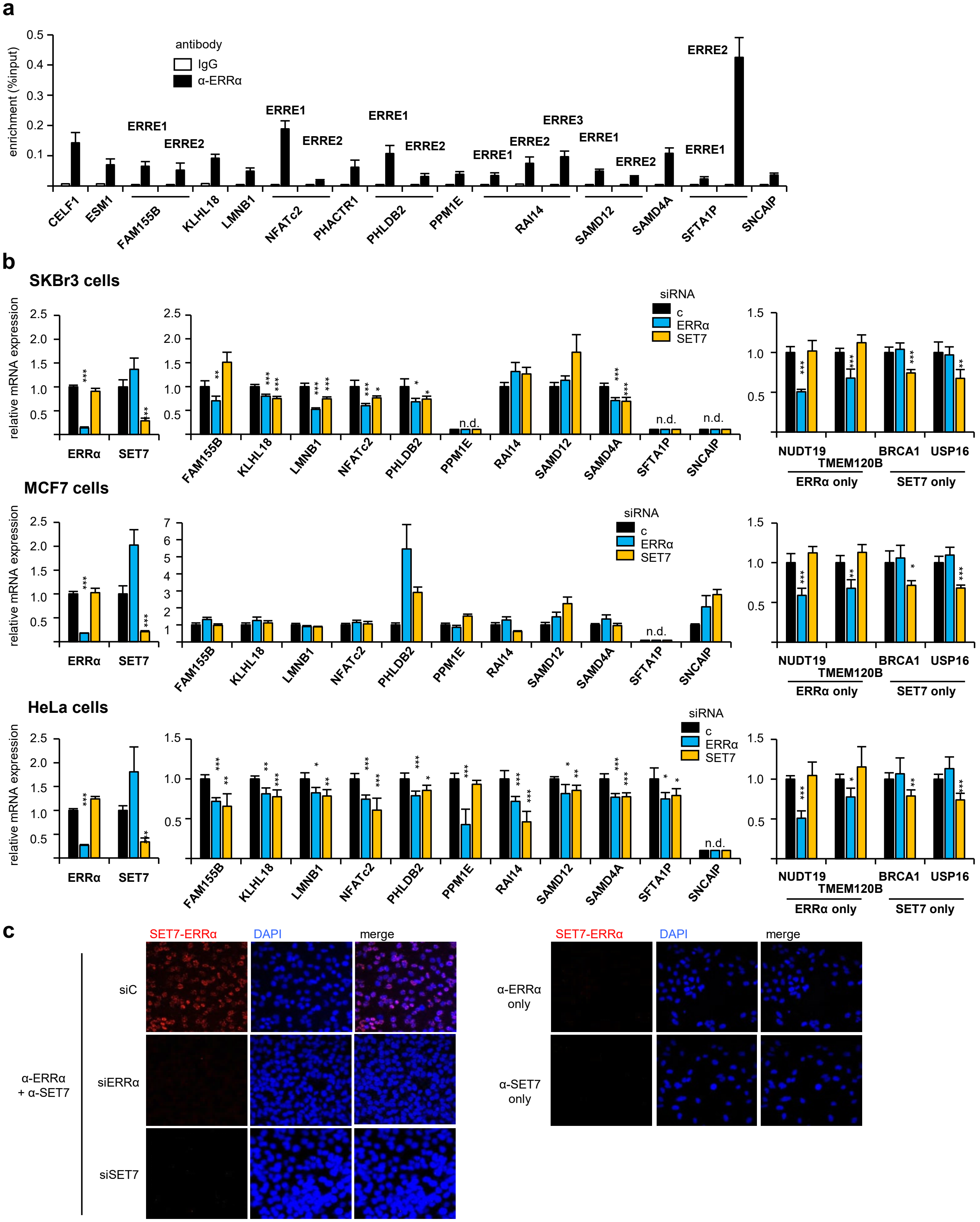

Figure S5. Validation of ERRα-SET7 target genes in MDA-MB-231 cells and regulation of their expression by ERRα and SET7 in SKBr3, MCF7 and HeLa cells. a. Binding of ERRα to genomic sequences identified by ChIP-Seq was confirmed by ChIP-qPCR experiments performed on independent samples. Percent enrichments after IP with anti-ERRα antibody or IgG were measured by qPCR amplifying a region encompassing the identified ERRα response elements (ERREs). CETN3, DARS and intergenic represent genomic regions where no ERRα peak was detected in ChIP-Seq experiments and were used as negative controls. Values are expressed relative to input. Bars represent mean +/- sem of two independent experiments performed in duplicate. **b.** Expression of the indicated genes, ERRα-SET7 activated genes in MDA-MB-231 cells, after siERRα or siSET7 transfection in SKBr3, MCF7 or HeLa cells. n.d. not detectable. As evaluated by t-test, variations are not significant unless indicated by ***: $p<0.001$, **: $p<0.01$, *: $p<0.05$ vs si-c. **c.** Controls for the PLA experiments. Left panel: same as **Figure 6f** using anti-ERRα and anti-SET7 antibodies on MDA-MB-231 cells treated with the indicated siRNA. Right panel: same as **Figure 6f** using a single antibody as indicated.

Figure S6

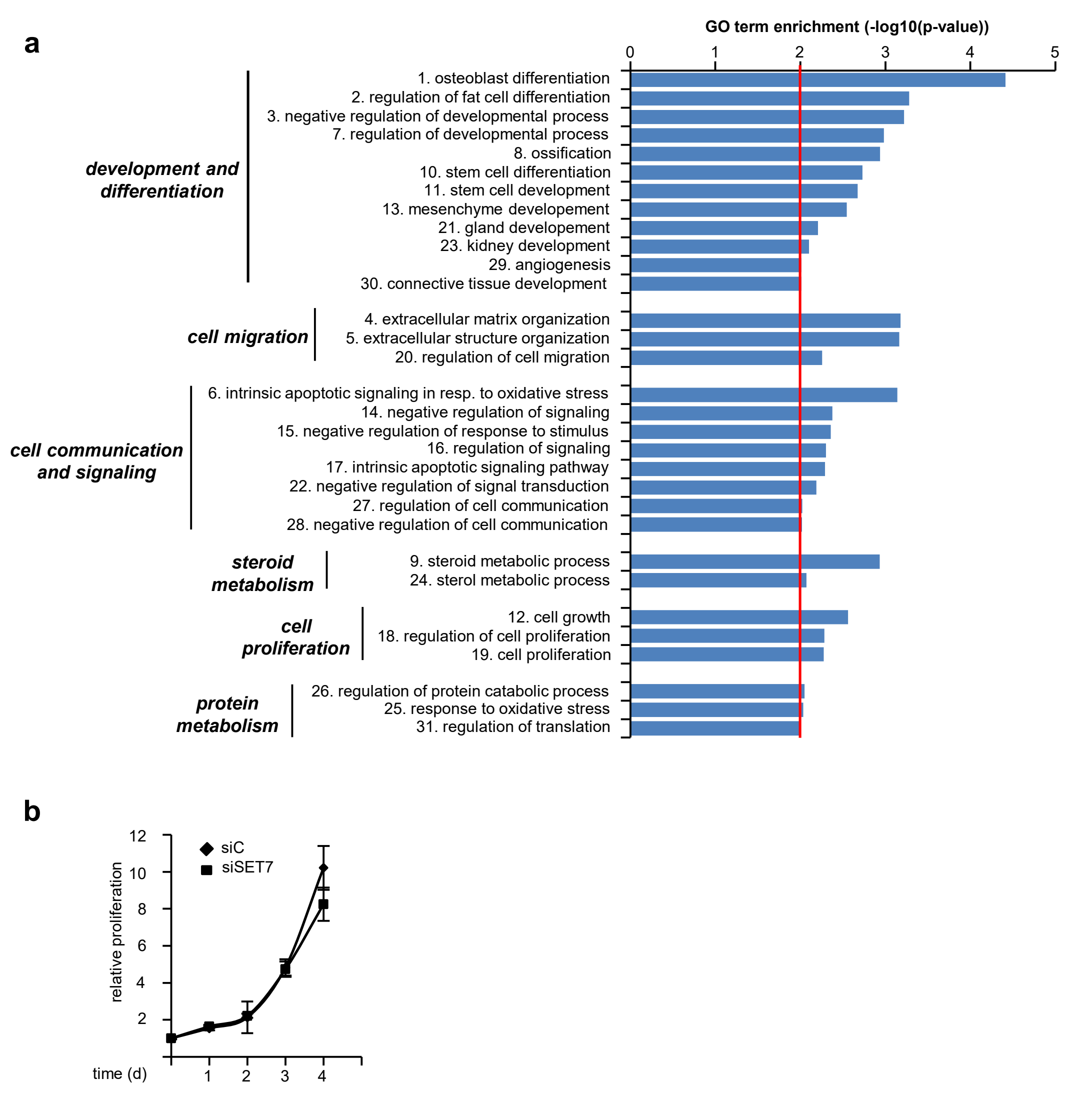

Figure S6. Functional involvement of SET7 in MDA-MB-231 cells.
a. Extended list of enriched GO terms in ERRα-SET7 common targets. Numbers correspond to the representation in **Figure 7d**. –log(P value) is indicated as enrichment level. Enrichment >2 (red line) were considered significant.
b. Analysis of MDA-MB-231 cell proliferation after transfection with sicontrol (siC) or siSET7. Relative cell numbers are indicated. Values are mean +/- sem of two independent experiments performed in triplicate. Differences at a given time point are not significant.

Figure S7

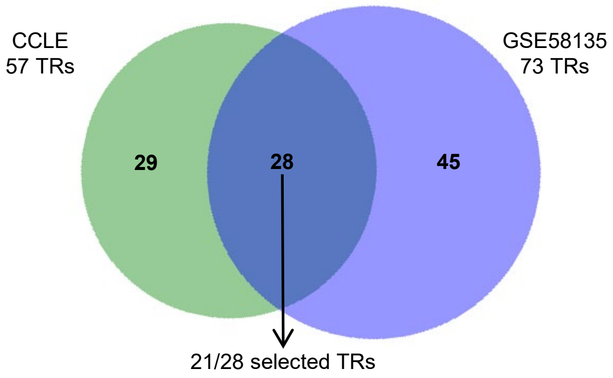

Common coTR	mean gene prop	Common coTR	mean gene prop
DDX21	0.53464	***ENO1***	0.23961
MYBBP1A	0.50392	***ELP3***	0.23294
NOC2L	0.48457	***NFKB1***	0.22207
NCL	0.39205	*EPAS1*	0.21788
SUPT16H	0.36947	***APEX1***	0.211
PPARGC1B	0.36947	*ZNF512B*	0.2032
MEN1	0.34594	*CEBPB*	0.20171
PA2G4	0.32652	*ZNF143*	0.1927
GMEB2	0.30098	*STRAP*	0.18572
SETD7	0.2636	*WDR5*	0.18465
WAC	0.26141	*ID2*	0.18248
ZNF710	0.24949	*WDR77*	0.17379
SNAPC4	0.24931	*GTF3A*	0.168
RLIM	0.24201	*BRD3*	0.13193

Figure S7. New computations after adding *NCOA1-2-3, PPARGC1A, PPARGC1B, PROX1* and *NRIP1* to the 318 TRs of our short list. These 7 factors have been shown to interact with ERRα and modulate its transcriptional activities. Using CCLE and GSE58135 data, expression modeling over 10 replicates were performed. 28 TRs common to the 2 datasets were obtained (including 21 with mean prop <0,2 in red), among which 19 TRs (green background) were among the 27 previously identified coTRs. 14/21 TRs were part of the 24 previously selected TRs (in bold), including *DDX21, MYBBP1A*, *SETD7*, and *NFKB1*.

PPARGC1B is identified prominently, despite its very low expression. None of the other added TRs (*NCOA1-2-3, PPARGC1A, PROX1* and *NRIP1)* were identified here.

References for *PROX1* and *NRIP1*:

- Charest-Marcotte, A. *et al.* The homeobox protein Prox1 is a negative modulator of ERRa/PGC-1a bioenergetic functions. *Genes Dev* 24, 537-542 (2010).
- Nichol, D., Christian, M., Steel, J.H., White, R. & Parker, M.G. RIP140 expression is stimulated by estrogen-related receptor alpha during adipogenesis. *J Biol Chem* 281, 32140-32147 (2006).

Figure S8

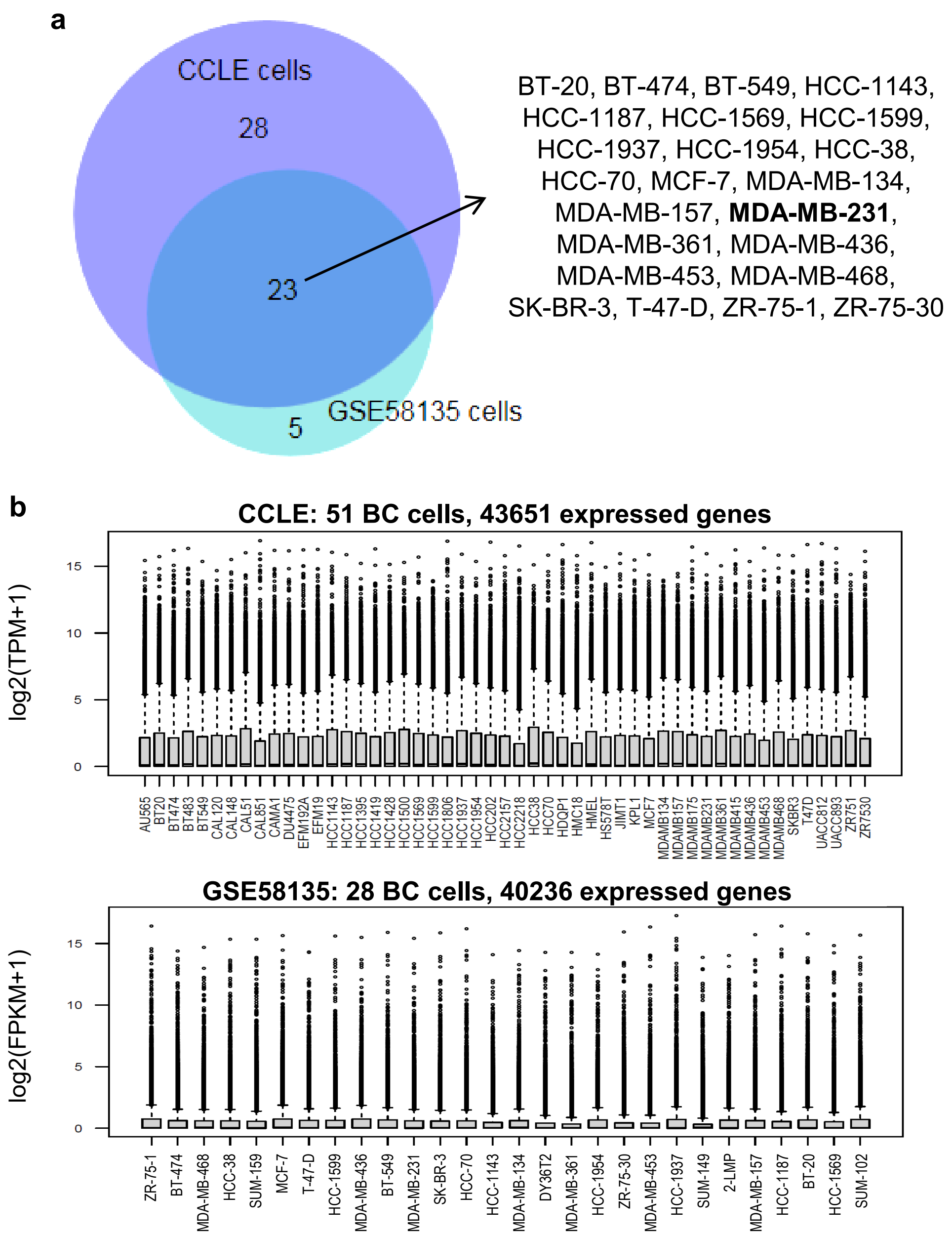

Figure S8. Breast cancer cell lines and gene expression across cells.
a. Venn diagram comparing the BC cell lines examined in the CCLE and GSE58135 studies (1, 2). They shared 23 cell lines including MDA-MB-231, from which the ERRα target genes were derived, and MCF-7 cells.
b. Box plots of each sample showing expression distribution of expressed genes in each dataset. In the CCLE dataset, 43651 out of 57820 genes were expressed in at least one cell type. For the GSE58135 dataset, only expression values with status OK were taken into account, other ones (LOWDATA or FAIL status) were replaced by NA, resulting in 40236 out of 51652 expressed genes.

1. Ghandi,M., Huang,F.W., Jané-Valbuena,J., Kryukov,G.V., Lo,C.C., McDonald,E.R., Barretina,J., Gelfand,E.T., Bielski,C.M., Li,H., *et al.* (2019) Next-generation characterization of the Cancer Cell Line Encyclopedia. *Nature*, **569**, 503–508.
2. Varley,K.E., Gertz,J., Roberts,B.S., Davis,N.S., Bowling,K.M., Kirby,M.K., Nesmith,A.S., Oliver,P.G., Grizzle,W.E., Forero,A., *et al.* (2014) Recurrent read-through fusion transcripts in breast cancer. *Breast Cancer Res Treat*, **146**, 287–297.

Figure S9

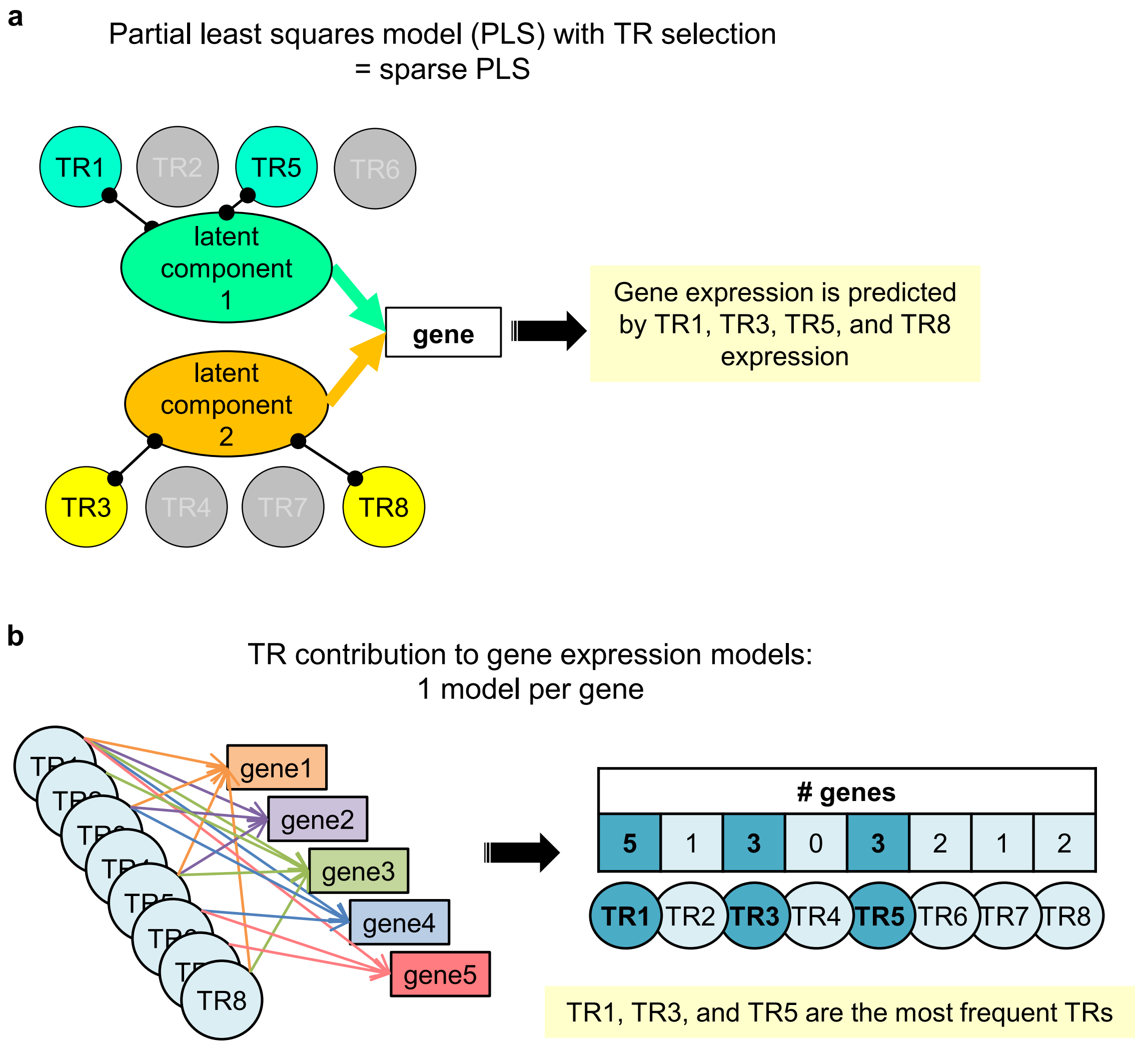

Figure S9. Sparse partial least square (sPLS) modeling.
a. General principle of the sPLS modeling. The model built to explain the expression of one gene uses latent components made of linear combination of transcriptional regulators (TRs) selected by the modeling algorithm.
b. Models computed for a set of genes used the same set of TRs. For each TR, the proposed procedure gives the number of genes for which the TR is included in the computed model to select the most frequently detected TRs.

Figure S10

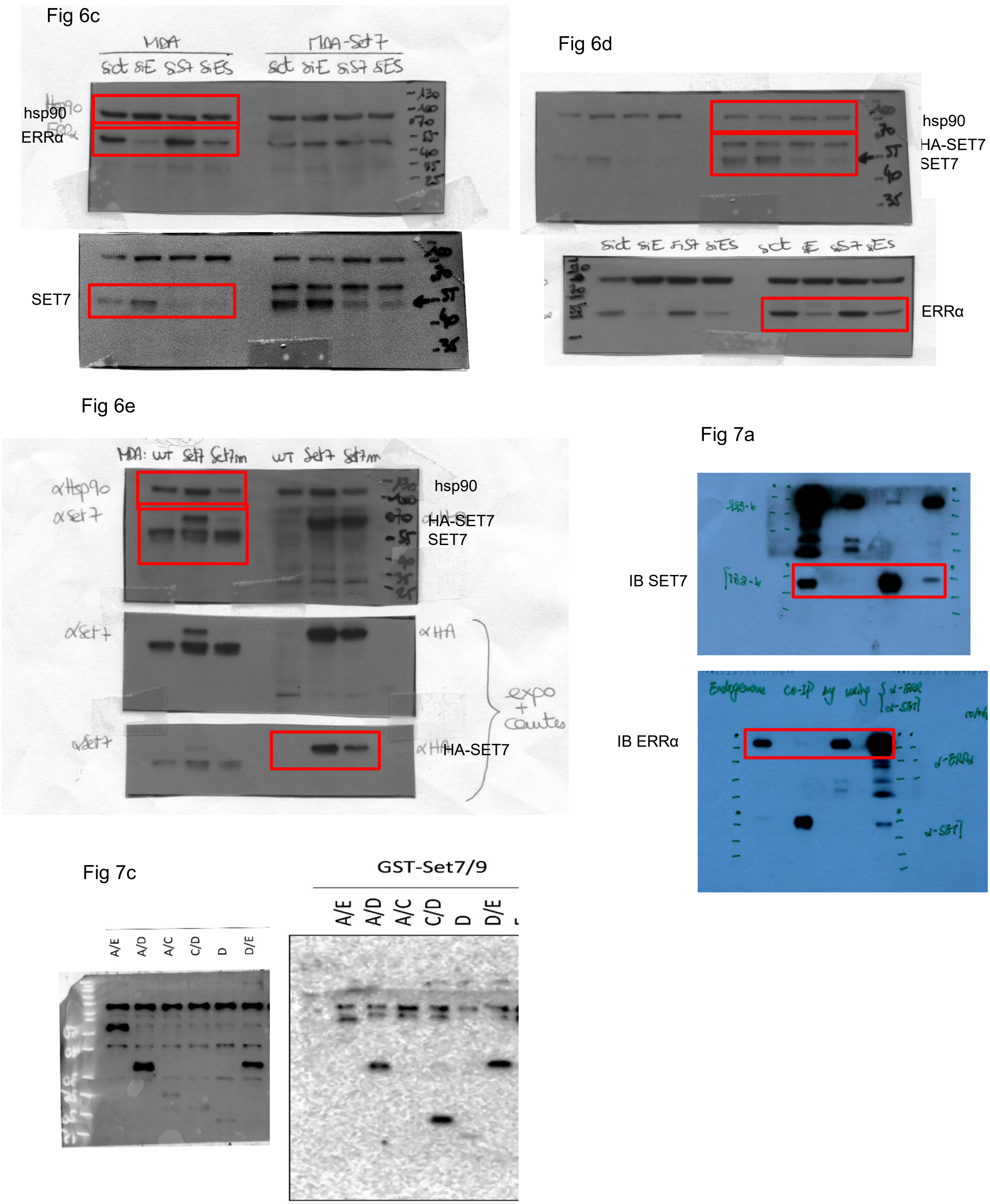

Figure 10. Full-length images of Western-blots presented in Figure 6 and 7.
The cropped images are shown with red rectangles and the corresponding figure panel is indicated. For Fig. 6e and 7a, different exposures of the same blot are displayed. Contrast was modified on Fig. 7c, so as to show the blot edges.
